# Single electrode dynamic clamp with StdpC

**DOI:** 10.1016/j.jneumeth.2012.08.003

**Published:** 2012-10-15

**Authors:** David Samu, Vincenzo Marra, Ildiko Kemenes, Michael Crossley, György Kemenes, Kevin Staras, Thomas Nowotny

**Affiliations:** a School of Engineering and Informatics, University of Sussex, Falmer, Brighton BN1 9QJ, UK; b School of Life Sciences, University of Sussex, Falmer, Brighton BN1 9QG, UK

**Keywords:** Electrophysiology, Dynamic clamp, Hybrid system, Current injection artifacts, Active electrode compensation, Digital compensation, Bridge balance, Capacitance compensation

## Abstract

Dynamic clamp is a powerful approach for electrophysiological investigations allowing researchers to introduce artificial electrical components into target neurons to simulate ionic conductances, chemical or electrotonic inputs or connections to other cells. Due to the rapidly changing and potentially large current injections during dynamic clamp, problematic voltage artifacts appear on the electrode used to inject dynamic clamp currents into a target neuron. Dynamic clamp experiments, therefore, typically use two separate electrodes in the same cell, one for recording membrane potential and one for injecting currents. The requirement for two independent electrodes has been a limiting factor for the use of dynamic clamp in applications where dual recordings of this kind are difficult or impossible to achieve. The recent development of an active electrode compensation (AEC) method has overcome some of these prior limitations, permitting artifact-free dynamic clamp experimentation with a single electrode. Here we describe an AEC method for the free dynamic clamp software StdpC. The AEC component of StdpC is the first such system implemented for the use of non-expert users and comes with a set of semi-automated configuration and calibration procedures that facilitate its use. We briefly introduce the AEC method and its implementation in StdpC and then validate it with an electronic model cell and in two different biological preparations.

## Highlights

► A novel implementation of active electrode compensation is introduced for the soft-real time dynamic clamp software StdpC to allow artefact-free dynamic clamp with a single, potentially high impedance, electrode. ► The method and its implementation are tested on an electronic model cell and in two different electrophysiological preparations. ► For the first time, active electrode compensation has been successfully verified with a second, independent electrode both in an electronic substitute cell and in a biological neuron. ► The new method is provided as part of the free software StdpC and comes with semi-automated calibration methods that allow its application by non-expert users.

## 1 Introduction

The dynamic clamp protocol was introduced independently by Robinson and Kawai (1993) and Sharp et al. (1993). Initially it was mainly used to inject artificial conductances into neurons to mimic the effect of voltage-gated ion channels and synaptic inputs from other neurons, but in the last twenty years it has become a versatile electrophysiological technique with a diverse application domain (Prinz et al., 2004). For instance, dynamic clamping has also been employed to fully control cell activity, so-called pattern clamp (Szücs et al., 2001) and even to simulate synaptic plasticity (Nowotny et al., 2003, 2006). Dynamic clamp systems have furthermore proved useful for experimental automation to implement complex and precisely timed protocols of interaction with neurons (Nowotny et al., 2006; Szücs et al., 2010).

Any dynamic clamp experimentation is based on a fast “dynamic clamp cycle” in which the membrane potential of a neuron is measured, the current that would flow through a model ionic channel at the measured potential is calculated, and this current is then injected into the same neuron. If the same electrode is used for membrane potential measurement and current injection, the resistance and capacitance of the electrode will cause current injection artifacts in the membrane potential measurement. In particular, the Ohmic resistance of the electrode will cause a voltage drop along the electrode proportional to the injected current while the capacitance will generate voltage drops proportional to the time derivative of the injected current which can be quite large for the rapidly changing current injections that are typical for dynamic clamp. Until recently, the most satisfactory solution to this problem has been to use separate electrodes for current injection and measurement. The electrode used for membrane potential measurements then is independent from the current injections and can provide unbiased measurements. However, inserting two electrodes into all target cells of interest can be technically difficult and in the case of many smaller neurons, especially in mammalian preparations, a major constraint on experimental success. This has limited the application of dynamic clamp as a research method and, to some extent, explains why it has not yet become a standard electrophysiological tool. Two-electrode dynamic clamp has an additional limitation in larger cells which are not electrically compact: the non-correspondence of the membrane potentials at the sites of the voltage measurement and current injection electrodes can introduce errors in dynamic clamp calculations.

In cases where only a single electrode can be inserted into a cell and is used for both current injection and recording of the membrane potential, the electrode artifact is usually compensated by bridge balance circuits and capacitance compensation circuits of electrophysiology amplifiers or by using a discontinuous current clamp mode (DCC) that is usually available with the microelectrode amplifier (see The Axon Guide (Molecular Devices, 2008) for a detailed discussion). However, these approaches have specific limitations. Bridge balancing only compensates for a simplified RC circuit model of the electrode, which is an oversimplification that is unlikely to fully remove current injection artifacts from the measured membrane potential signal in particular because the fast current transients, that are typical in dynamic clamp, emphasize the deviations of the real electrode from the simplified RC model (see Section 5 below for a concrete example). Furthermore, the remaining artifacts are particularly problematic in dynamic clamp because it operates in a fast feedback loop and any noise or artifacts on the voltage signal are fed back, potentially amplified, into the cells through subsequent current injections.

DCC on the other hand requires the use of an electrode that is an order of two magnitudes faster than the observed cellular phenomena, which makes it inappropriate for most dynamic clamp experiments that are aiming to simulate rapidly changing trans-membrane currents. Moreover, DCC introduces high frequency noise into the amplifier circuitry which is often eliminated by low-pass filtering. Although this solution is acceptable for most other applications, filtering the input to the dynamic clamp system makes it less sensitive to fast changes in the membrane potential which again limits the timescales of the simulated currents.

The active electrode compensation (AEC) method introduced by Brette et al. (2007, 2008) takes a radically different approach. AEC uses a digital model of the electrode, implemented in a computer, with parameters obtained during a short calibration phase, and estimates the voltage artifact of the electrode based on this calibrated model entirely within the computer software. The estimated artifact is calculated and digitally subtracted from the measured voltage values in every dynamic clamp cycle. The underlying model for the current injection artifact of the electrode is that of a time-invariant linear filter *F* applied to the time series of ‘recently’ injected current values *I*(*t*). This filter is fully characterized by the convolution of the injected current signal *I*(*t*) with the so-call electrode kernel, *K*_*e*_, see Eq. (1) below.

**Fig. 1:**
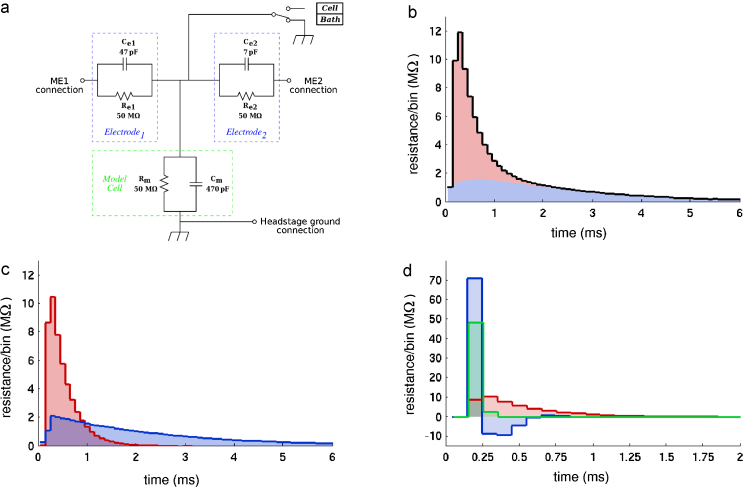
Illustration of the electrode kernel concept. (a) Electronic circuit (“model cell”) used for the first series of verification experiments. The three RC circuits represent two electrodes and the membrane of a neuron respectively. (b) Estimated full kernel *K* for the model cell. The colours indicate the contributions from the electrode kernel *K*_*e*_ (red) and the filtered membrane kernel *K*_*m*_ * *K*_*e*_/∫*K*_*e*_ (blue). (c) Electrode kernel *K*_*e*_ (red) and membrane kernel *K*_*m*_ (blue) after numerical separation. (d) Effect of capacitance compensation of slow electrodes by the amplifier. Without capacitance compensation (red) the estimated electrode kernel of a slow electrode is too broad, while too much compensation (blue) introduces oscillatory instabilities. The green kernel is estimated at an optimal level of capacitance compensation.

The electrode kernel is estimated during calibration from the measured voltage responses to low amplitude noise injections. In this process the responses due to the neuron membrane (*K*_*m*_) are separated from the electrode artifacts based on their different time scales. Fig. 1 illustrates typical kernel estimates as they occur on a substitute model cell.

Being a generalization of the traditional RC electrode models, AEC promises to deliver higher fidelity electrode compensation than the classic bridge balance and capacitance compensation techniques. For further details of the underlying mathematical theory of this method see (Brette et al., 2007, 2008) and the corresponding supplementary material.

The method as originally introduced by Brette et al. (2007, 2008) depends on a fixed time-step, real time dynamic clamp platform. StdpC is based on the popular but non-real time Windows operating system that can only provide a soft real-time environment, and StdpC operates with variable time-steps, in contrast to many other dynamic clamp systems that are based on real time operating systems (Dorval et al., 2001; Butera et al., 2001; Culianu and Christini, 2003; Bettencourt et al., 2008; Preyer and Butera, 2009) or dedicated real-time hardware subsystems (Kullmann et al., 2004; Robinson and Kawai, 1993; Robinson, 2008). In order to use the AEC technique in such a soft real-time environment, we have created a soft real-time implementation of active electrode compensation within StdpC, which we present here. Our novel implementation includes semi-automated configuration and calibration methods. Specific electrode- and cell-dependent parameters are set automatically by the software during the semi-automatic calibration procedure and do not require adjustments by an expert user. However, if desired, AEC settings can be adjusted effortlessly by expert users through the graphical user interface.

This paper provides a detailed description of how dynamic clamp experiments can be performed with StdpC using our novel AEC method, independently of the specific preparation and electrophysiological phenomena under investigation. We provide sample data from an artificial “model cell” that for the first time directly validates the AEC method (and its implementation in StdpC) against two-electrode measurements. Importantly, we then also show results from two contrasting experimental preparations: invertebrate neurons recorded *in situ* in the intact nervous system and cultured rat hippocampal cells recorded *in vitro*.

## 2 Materials and methods

### 2.1 Biological preparations and experimental procedures

#### 2.1.1 Preparations

Two types of preparations were used to develop and explore single electrode dynamic clamp with StdpC.

The first type of preparation was the intact cerebral ganglia of the pond snail *Lymnaea stagnalis*, a well-known model for investigating the properties and functions of identified neurons (Kemenes and Benjamin, 2009). We specifically targeted the Cerebral Giant Cells (CGCs) in our experiments. These cells are extremely well-identified, from the level of their membrane conductances (Staras et al., 2002) through their axonal projections and firing properties (McCrohan and Benjamin, 1980) to their synaptic connections with other neurons (McCrohan and Benjamin, 1980). Recently, the CGCs also have been shown to play a key role in long-term memory forming after classical conditioning (Kemenes et al., 2006; Nikitin et al., 2008), making them very useful models for investigations of how changes in the properties of individual neurons, controlled by dynamic clamp, can shape memory traces at the circuit level. The ability to use a single-rather than two-electrode dynamic clamp method to alter the properties of this cell is advantageous because the other electrode can then be used to record from a synaptic follower cell at the same time as manipulating the CGC. Due to its large size (exceeding 100 μm in diameter) the use of this cell also was advantageous during the development of our single-electrode DC method for technical reasons. Because it was possible to insert two electrodes into the cell, we could make direct comparisons between the application of single-electrode versus two-electrode DC in the same cell. Also, the large size of this cell allowed us to test the limits of our single-electrode DC method.

The second type of preparation was hippocampal neuronal cell cultures from P0 rats. We chose this system to demonstrate the particular challenges associated with electrophysiological recording from small mammalian neurons where two electrode recording is not a viable option. Dissociated neurons are plated onto an astrocyte feeder layer to form mixed cell networks. This culture preparation is an established and standard model for examining ionic mechanisms of neuronal excitability and exploring network connectivity properties in mammalian neurons. Using AEC we were able to introduce large currents and gain an accurate read-out of the membrane potential without the need for two electrodes. In recent years, the membrane potential of the presynaptic cell has been shown to play a role in synaptic transmission beyond the simple generation of the action potential, a phenomenon referred to as hybrid analog and digital signalling (Clark and Husser, 2006). The first studies to show direct evidence for hybrid signalling used extremely challenging simultaneous recordings from the soma and the axon/bouton (Alle and Geiger, 2006; Shu et al., 2006). Due to the low threshold for action potential in the axon initial segment (Kole et al., 2006) simple current steps proved inadequate for achieving large sub-threshold changes in potential at the soma with tight temporal control. Here, by injecting a computer generated pattern of currents to produce a dynamically controlled change in voltage (pattern clamp), we were able to produce rapid sub-threshold changes in membrane potential at the soma, an important tool for studying the effect of membrane potential in synaptic transmission.

#### 2.1.2 Intracellular recordings

Brains dissected from 2-month-old *Lymnaea stagnalis* were kept in *Lymnaea* saline solution containing in mM: 50 NaCl, 1.6 KCl, 3.5 MgCl_2_, 2 CaCl_2_, 10 HEPES buffer in water (pH 7.9). The outer connective tissue was removed from the ganglia before recording to facilitate microelectrode impalement. Intracellular recording and current injections were carried out using sharp electrodes produced from 2 mm outer diameter thick-walled capillaries (Harvard Apparatus) filled with 2 M potassium acetate, with a final resistance of 20 MΩ. Identified neurons (the cerebral giant cells, CGCs) were impaled and recorded using an Axoclamp 2B amplifier (Molecular Devices). Impalement was further facilitated by a brief overcompensation of the capacitance neutralization circuit. Cell health and quality of the impalement was assessed by the experimenter before proceeding to the active electrode compensation.

#### 2.1.3 Whole-cell patch clamp recordings

Hippocampal neuronal cell cultures from P0 rats were obtained following protocols described in Darcy et al. (2006). Electrophysiological recordings were carried out in extracellular bath solution containing in mM: 137 NaCl, 5 KCl, 2.5 CaCl_2_, 1 MgCl_2_, 10 d-glucose, 5 HEPES buffer 20 μM 6-cyano-7-nitroquinoxaline-2,3-dione (CNQX), 50 μM d(−)-2-amino-5-phosphonovaleric acid (AP5) in water (pH 7.35). Patch electrodes used to obtain whole-cell recordings were obtained by 1.5 mm OD thick wall borosilicate glass capillaries filled with intracellular solution containing in mM: 115 KMeSO_4_, 5 KCl, 4 NaCl, 0.5 CaCl_2_, 10 creatine phosphate, 2 MgATP, 2 Na_2_ATP, Na_3_GTP, 10 glutamic acid, 10 HEPES. The electrode's final resistance was 3–5 MΩ with a total access resistance lower than 30 MΩ.

### 2.2 Dynamic clamp hardware and software setup

StdpC is available from sourceforge at http://sourceforge.net/projects/stdpc/. All versions from 2011 onwards contain the AEC feature, located in Config → Electrode setup. The general hardware and software setup of StdpC has been described in detail previously (Nowotny et al., 2006; Kemenes et al., 2011) and remains the same when using AEC. StdpC is supported in Windows XP and Windows 7, and can be used on any recent PC, preferably with a multi-core CPU. The software works with any National Instruments (NI) data acquisition board supporting the NIDAQmx interface (essentially all modern NI boards). However, we discourage the use of USB devices as they have longer communication latencies and hence lead to low update frequencies of the dynamic clamp cycle. StdpC also continues to support the original DigiData 1200(A) boards (Axon Instruments, part of Danaher) even though the quality of dynamic clamp is higher with the modern 16 bit NI boards.

All experiments with StdpC are performed in current clamp mode. When using AEC, as described here, the bridge balance mode of the amplifier needs to be turned off (set to 0 level), continuous injection/recoding mode must be used, and the capacitance neutralization feature of the amplifier needs to be 0 (but also see below).

The input/output channel configuration in StdpC is set up as previously described (Kemenes et al., 2011), however, setting the correct gain factors and the most appropriate acquisition ranges do need additional consideration when using the new AEC method in StdpC. Due to the digital nature of StdpC's built-in electrode compensation technique, the directly recorded voltage values will be uncompensated and hence contain the electrode artifact. For correct operation the DAQ must be able to digitize the full range of observed voltage signals of membrane potential plus current injection artifact on its input channels. This range can be considerably larger than the expected effective electrophysiological signal range. A rough estimate of the maximum expected electrode artifact can be obtained using Ohm's law, giving a range of [*I*_min_*R*_*e*_, *I*_max_*R*_*e*_], where *R*_*e*_ is the electrode resistance and *I*_min_ and *I*_max_ are the expected lowest (most negative) and highest injected current amplitudes. Note that the artifact can be considerably larger than the corrected electrophysiological membrane potential.

## 3 Soft real time implementation of AEC

The AEC method for electrode compensation was introduced by Brette et al. (2007, 2008) as an alternative to the standard methods of bridge balance, capacitance compensation and discontinuous current clamp recording and current injection. Here we give a brief overview of the method and describe our modification to the soft real-time environment of StdpC. The reader is referred to the original publications (Brette et al., 2007, 2008) and their detailed supplemental material for additional theoretical details.

The fundamental assumption underlying AEC is that the current injection artifact observed at a micro-electrode can generally be expressed as a convolution of the injected current signal *I*(*t*) and a constant kernel function *K*_*e*_,

(1) $$V_{e}(t)=(K_{e}*I)(t)=\int_{0}^{T}K_{e}(x)\;I(t-x)\;\mathrm{dx}$$

The electrode kernel *K*_*e*_ can be estimated during a calibration phase from recording the voltage response to a known random series of current steps at high frequency (10 kHz). The procedure of kernel estimation is illustrated in Fig. 1. The electrode properties can change dramatically during cell impalement or patching. The calibration of the electrode kernel, therefore, has to be performed after the contact with the cell of interest is established. In this case the overall voltage response is a combination of the cell response and the electrode response, *V* = (*K*_*m*_ * *K*_*e*_/∫*K*_*e*_) + *K*_*e*_ * *I*, where the convolution with *K*_*e*_/∫*K*_*e*_ takes account of additional filtering of the membrane response through the electrode. The two contributions of the passive membrane and the electrode can be separated numerically if the time constants of the electrode and the membrane are sufficiently different. In practice, good AEC quality can be achieved for *τ*_*m*_/*τ*_*e*_ > 10, where *τ*_*m*_ and *τ*_*e*_ are the characteristic time constants of the membrane and the electrode respectively. In order to improve (lower) the effective electrode time constant the amplifier's capacitance compensation system can be used (see StdpC's User Manual (supplementary material) and (Brette et al., 2008) supplementary information for details).

AEC was developed for a real-time environment with guaranteed constant time steps in both calibration and application of the artifact compensation. In the implementation within StdpC, the calibration is performed in tightly controlled pseudo-realtime with almost constant Δ*t*. The quality of this procedure is transparent to the user (see below and Fig. 3, yellow marks). The actual dynamic clamp cycle is run in soft realtime with potentially quite variable time steps Δ*t*_*n*_. In order to use the AEC compensation in this environment we have adopted an interpolation method for calculating the convolution of the fixed time-step estimated electrode kernel *K*_*e*_ and the variable time step current injection history *I*(*t*_*n*_). The basic idea behind our approach is that for the calculation of the *V*_*e*_(*t*_*n*_) electrode artifact, the convolution

(2) $$V_{e}(t_{n})=\sum_{i=1}^{N}K_{e}(t_{i})\;I(t_{n-i})\;\Delta t$$

can be generalized for non-equal Δ*t*_*j*_ = *t*_*j*_ − *t*_*j*−1_ time steps, as long as *K*_*e*_ and *I* are given at the same times *t*_*n*_. More specifically, the equally sampled kernel, obtained during the calibration phase and stored as the “base kernel” during clamping, can be transformed at each clamping cycle into an inhomogeneously sampled one, that matches the sampling times of the current vector *I*(*t*_*n*_) which is dictated by the operating system. To this end, as the dynamic clamp experiment proceeds, both the injected currents and the sampling times are stored within an *L*_*e*_ wide sliding window, which is given by the kernel width and the recently observed sampling times. Within each clamping cycle, the “base kernel” (the kernel with constant time steps obtained during calibration) is fitted onto the most recent sampling times by the following interpolation: (1) all bins of the base kernel that fall within the same sampling interval of *I* are summed, and (2) bins of the base kernel that are on the border of the sampling intervals are split into two and distributed to the two adjacent intervals proportionally to the amount of overlap. Using this interpolation we obtain a transformed electrode kernel $K_{e}^{\mathrm{tr}}$ with sampling times identical to those of the injected current *I*. Then the generalized asynchronous convolution $V_{e}=K_{e}^{\mathrm{tr}}*I$ can be calculated.

All parts of the AEC method, including tests for its applicability for a given electrode/cell, the calibration phase, the kernel estimation and separation, and the actual compensation are implemented as an integral part of StdpC and are accessible through an intuitive graphical user dialog.

## 4 Using AEC in StdpC

**Fig. 2:**
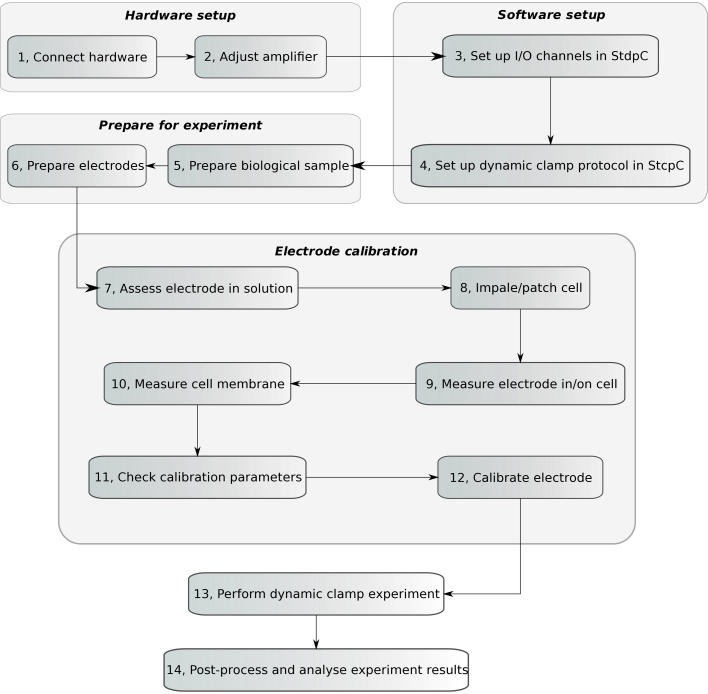
Schematic diagram of the general steps of an AEC compensated dynamic clamp experiment using StdpC. The six main parts of the procedure are: hardware setup, software setup, experiment preparation, electrode calibration, performing experiment, and finally, result analysis. A detailed explanation of these steps is given in Sections 3 and 4.

A general AEC enabled electrophysiological experiment using StdpC can be divided into six main parts: hardware setup, software setup, experiment preparation, electrode calibration, performing the experiment, and finally, analyzing the result (Fig. 2). The first, third and the fifth phases are only slightly affected by StdpC and the AEC technique (although carefully chosen I/O channels, electrolytes and supervision during recording are essential, just as when using any other dynamic clamp system). Setting up StdpC's channels and the actual protocol (part two), however, as well as the calibration of the electrodes (part four) are particular to the system introduced in this article.

As explained above, the method of active electrode compensation (AEC) requires a few important steps before the actual experimentation can begin. At the beginning of an experiment using AEC, the properties of the electrodes need to be assessed in the bath solution to determine whether the electrode (and amplifier, filters) are appropriate for AEC. This comprises three key measurements:

1. Measuring the mean resistance and assessing that it matches the resistance expected for the type of electrode used based on information in the literature or prior experimentation.
2. Establishing that the variation of the steady state resistance of the electrode does not vary significantly with different (moderate) levels of injected DC current (*σ*_*R*_/*μ*_*R*_< 15 %, where *σ*_*R*_ denotes the standard deviation and *μ*_*R*_ the mean of the electrode resistance as observed from a set of moderate current injections). In other words we need to establish that the electrode can be considered as a linear (Ohmic) resistor for which the voltage is a linear function of the injected steady state current (Fig. 1A). The approximate linearity of the electrode is a prerequisite for AEC (see above).
3. Measuring the time constant (RC constant) of the electrode and establishing that it is at least 10× smaller than the expected passive membrane time constant (RC constant of the membrane), i.e., that the responses of the electrode are at least 10× faster than those of the passive cell membrane. This generally equates to the constraint that the electrode time constant is less than 0.5–1 ms. In cases where the electrode is found to be too slow, one can use the capacitance neutralization feature of the amplifier to decrease the effective electrode capacitance and thus increase electrode speed (see Fig. 1d and the StdpC's User Manual). The more principled solution may however be to replace the electrode for a faster one.

StdpC's Electrode Compensation dialog window offers convenient tools to perform these three control checks, as well as the actual AEC-specific electrode calibration that has to be performed once these checks have been completed successfully.

**Fig. 3:**
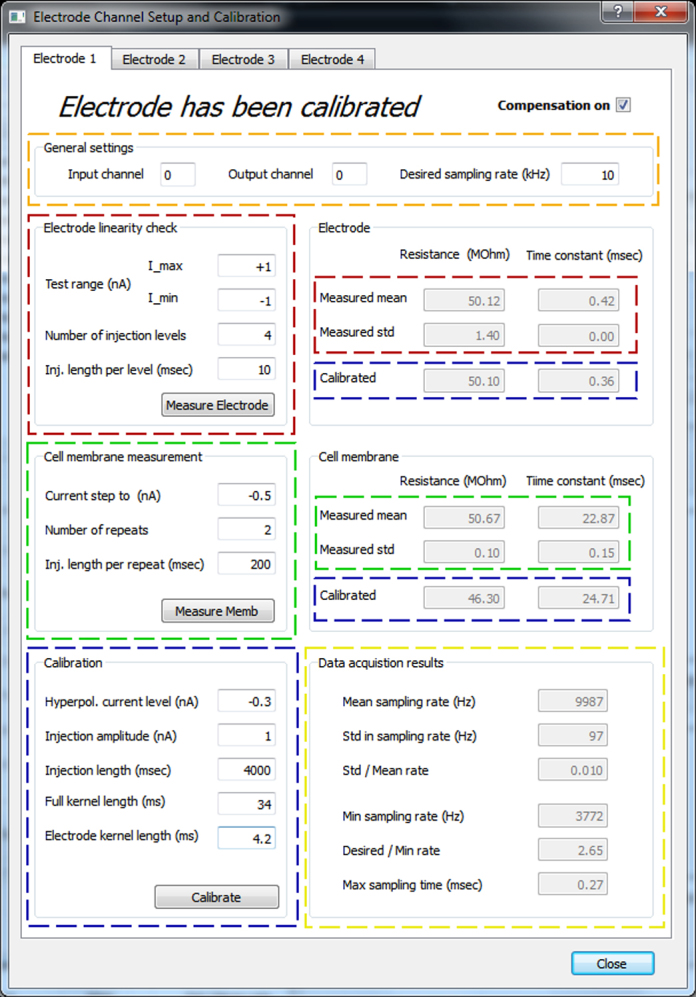
Electrode channel setup and calibration panel, showing the parts of the user interface corresponding to the main steps of the electrode calibration procedure. Five main parts are highlighted: gold: electrode setup, red: electrode measurement, both in bath and in/on a cell, green: cell membrane measurement, only after impalement/patching, blue: calibration utilities, yellow: for displaying result on data acquisition timing. Editable text fields on the top and left side of the panel have white background, while the gray shaded fields on the right side are not editable and display information only. Each function on the left side (electrode linearity check, cell membrane measurement, and calibration) can be initiated by its corresponding button, and the obtained results are displayed in the information fields on the right side, highlighted with the same colour. After triggering any of the three processes, the data acquisition results subpanel is updated as well in order to allow the user to assess the stability of the measurement/calibration. (For interpretation of the references to colour in this figure legend, the reader is referred to the web version of the article.)

The linearity of the electrode is assessed by measuring its steady state resistance at various current levels, typically within the expected domain of the subsequent experiment. To perform this measurement the experimenter enters the maximal, minimal, and number of injected current levels, or accept the default parameters offered by the software. Clicking the “measure electrode” button StdpC performs the specified series of current injections and calculates the mean electrode resistance, its variation with current injection level and an estimated electrode time (RC) constant. The results are displayed on the right of the calibration toolbox (Fig. 3, marked in red) allowing the experimenter to judge the electrode quality and suitability according to the above criteria. Checking the electrode in solution prior to contacting the cell allows the experimenter to rule out inappropriate electrodes with minimal effort and damage caused to the cell.

After the described basic electrode tests are successfully completed with the electrode immersed in the external solution of the preparation, the actual experimentation can commence by establishing an intracellular or whole-cell patch-clamp recording from a target neuron. After impalement/patching, the electrode is measured again and re-assessed with respect to the three criteria described above while it is in/on the cell. The impalement or patching of a neuron usually alters some characteristics of the electrode (most likely its resistance), and the electrodes are only suitable if the necessary conditions described above are still fulfilled, i.e., if both the resistance's standard deviation and the time constant are low enough (but not necessary the same ones that were measured in solution).

We found that the duration of the electrode test measurement(s) at this step is critical. At this point the electrode is already in contact with the cell and it is thus essential not to use more prolonged current injections than necessary, in order to keep the evoked cell response at a minimum, and to reduce the chance of interfering spiking activity of the cell during electrode characterization. We found that 4–5 times the electrode time constant is a good rule of thumb for the appropriate duration of current injections.

Another consideration is to ensure that the electrode(s) has/have reached a stationary state, i.e., that resistance and time constant remain stationary before the actual electrode calibration for AEC is performed. To test the stationarity of electrodes the resistance and capacitance tests described above can be repeated up to 10×. If the properties of the electrode do not stabilize, the electrode needs to be replaced.

After the electrode tests have been successfully completed, the cell membrane properties for each electrode/neuron are measured in an analogous way. The time constant of the cell membrane is an important parameter in the electrode calibration process and can be measured using a second built-in function in StdpC (Fig. 3, marked in green). Appropriate injection duration for this measurement should be at least 5× the expected membrane time constant (normally between 5 ms and 100 ms).

During the measurement of cell membrane resistance we recommend hyperpolarizing the cell to avoid evoking action potentials. Any evoked spike can be disruptive during the measurement of passive membrane properties, just as much as during the measurement of the electrode properties discussed above. Hyperpolarization during cell membrane measurement can be achieved directly in StdpC by specifying negative current steps. If a cell demonstrates ongoing activity during the measurement of passive membrane properties this will be reflected in high standard deviations of the estimated membrane resistance and/or time constant. In this case the measurement needs to be repeated, potentially with stronger hyperpolarizing current.

After the basic electrode and cell calibrations, the actual AEC calibration is performed. Standard parameters for the AEC calibration method are automatically generated from the results of the above discussed electrode and cell membrane measurements, but the software permits experimenters to manually adjust these settings. The recommended duration for the calibration is 4–5 s in order to acquire a statistically significant number of samples. The particular amplitude of the probing current depends on the hardware configuration and the excitability of the cell, 0.5 nA is generally a good setting here. If more than about 5 action potentials occurred during the injection of probing current, it is recommended to repeat the calibration with a mild hyperpolarizing current. The remaining two parameters (“Full kernel length” and “Electrode kernel length”, see Fig. 3, marked in blue) are specific to the AEC electrode calibration technique and are automatically chosen by StdpC based on the initial measurements of electrode and cell properties. However, the software allows the advanced user to manually modify these values as well.

During the calibration of an electrode a randomized current signal is passed through the electrode and into the cell. It is recommended to monitor the recorded voltage on a separate recording computer to rule out calibration errors due to high intrinsic or evoked cell activity. A few action potentials are inevitable and have only a small effect, but more than 5 spikes are likely to deteriorate the quality of the calibration markedly (see above). After calibration, the previously obtained electrode properties (electrode resistance and time constant) can be compared to the values that are derived automatically during AEC calibration. If the values differ significantly it is an indication that the method will not work properly and calibration needs to be repeated. The troubleshooting section in StdpC's User Manual helps with resolving the causes of such a calibration failure. Note that the electrode and membrane resistances and time constants are derived quantities of the more detailed electrode and neuron membrane kernels (Fig. 3, marked in blue) which capture much more detailed information about the passive responses of membrane and electrode. In particular, electrodes can have quite different kernels while having the same time constant and resistance, indicating that the AEC model may provide significant improvements over the traditional RC model.

The workflow of the calibration method is summarized in Fig. 2. After the AEC calibration has been completed successfully, StdpC can be used with all its previously described (Nowotny et al., 2006; Kemenes et al., 2011) functions and the signals from all calibrated electrodes will automatically be digitally corrected for current injection artifacts.

## 5 Results

**Fig. 4:**
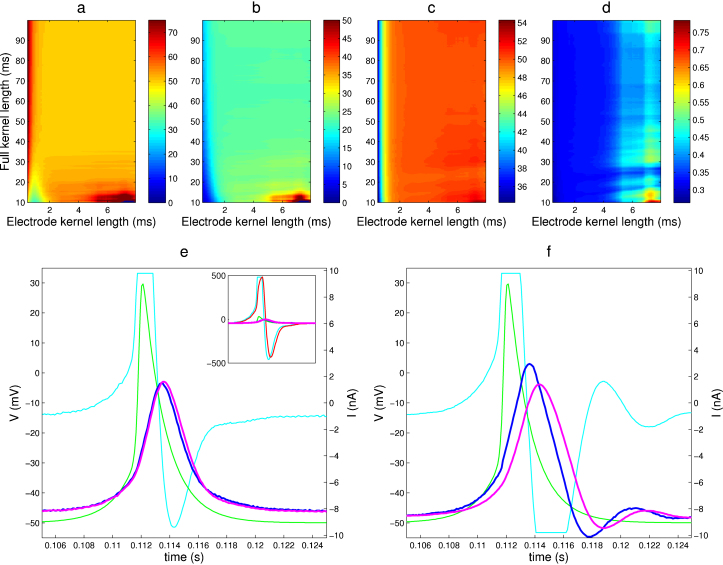
Demonstration of artifact compensation on a model cell. (a–d) AEC estimated circuit properties obtained from electrode calibration for a wide region of AEC's two crucial parameters (full kernel length and electrode kernel length, see Brette et al. (2008) for details). There is a large area in this parameter region where the true resistances and time constants are recovered correctly. (e–f) Comparison of AEC and bridge balance compensation for simulating a 500 nS gap junction (electrical synapse) from a simulated cell (spike generator) to the (physical) model cell. (e) Retrieved membrane potential using AEC and the AEC calculated electrode artifact (inset). (f) Results of using bridge balance compensation. Colour code: green: spike generator potential, cyan: injected current, red: calculated electrode artifact (*V*_*e*_), magenta: calculated membrane potential (*V*_raw_ − *V*_*e*_) in case of AEC (e), amplifier provided measurement of the membrane potential in case of bridge balance (g), blue: control (“true”) membrane potential on a separate channel. The bridge balance compensated signal is delayed and exhibits artifactual damped oscillations. This is a good example of how the errors in compensation are fed back into the system during dynamic clamp, leading to marked differences in the entire system's behavior. Model cell properties: model electrode: *R*_*e*_ = 50 MΩ, *τ*_*e*_ = 0.35 ms, model membrane: *R*_*m*_ = 50 MΩ, *τ*_*m*_ = 23 ms.

We tested the method and its implementation in StdpC in several stages. Fig. 4 shows the results of testing the AEC implementation in StdpC on a model cell (provided with the AxoClamp 2B amplifier used here) used for development and initial testing. The model cell itself is an electrical circuit comprising a parallel resistance–capacitance (RC) cell model. Two other, faster parallel RC components, modeling the electrodes, are connected serially to the two ends of the cell circuit (see Fig. 1A for a circuit diagram). In the first test, the model cell and one of the model electrodes were used to assess the stability of the implemented AEC calibration phase by exploring the joint parameter space of the two most important AEC calibration phase parameters, the so-called full kernel length and electrode kernel lengths. In the results, shown in Fig. 4a–d, we can identify a broad, common area (electrode kernel length: ∼2–5 ms, full kernel length: ∼30–100 ms), where the cell membrane and electrode properties (resistances and time constants) calculated during the AEC calibration match with the actual, known parameters of the model cell and model electrode. These results validate the robustness of the AEC calibration procedure in general and our implementation of it in particular.

In the next set of experiments, the StdpC's spike generator was set to act as an artificial presynaptic cell. It generated “spike-like” voltage waveforms that mimicked the shape of a generic action potential, with adjustable resting potential, spike height, spike width and timing parameters (see StdpC's user manual for details), and the resulting voltage waveform was connected presynaptically to the physical model cell through a simulated 500 nS gap junction (electrical/Ohmic synapse). The current, calculated by StdpC and generated by the Axoclamp microelectrode amplifier, entered the model cell through one of the model electrodes, implementing a configuration that is typical in real dynamic clamp experiments. At the same time the second electrode of the model cell was used to record the potential passively without any current injections to provide an unbiased control measurement. With this setup we were in a position to directly measure the quality of the AEC method in controlled conditions.

As expected we note a pronounced electrode artifact in the uncorrected signal of the current injection electrode, which in this case is about an order of magnitude larger than the response of the model cell “membrane” response (Fig. 4e inset). The large difference in amplitude mainly arises from the difference in response speed between the electrode and the cell (*τ*_*m*_/*τ*_*e*_ ≈ 70) and is amplified by the very strong coupling between the pre- and postsynaptic “cells” (500 nS). This example demonstrates the necessity of an increased range for the digital to analog converters on channels compensated by AEC (see Section 2) as the large artifactual voltage nevertheless has to be digitized correctly in order to then be digitally adjusted for the artifact. We then compared the results of different compensation techniques in this dynamic clamp configuration: StdpC's AEC (Fig. 4e) and the Axoclamp 2B microelectrode amplifier's bridge balance compensation (Fig. 4f). We observed no significant difference between the compensated and the control signal in the AEC case, while bridge balance compensation seems to lead to an undesirable time delay in the rising phase of the postsynaptic membrane potential during the course of a presynaptic action potential. The delay is introduced by the amplifier's bridge balance circuit, and while it can have negligible effects on the membrane potential signal in less demanding configurations, it begins to cause oscillatory after-effects on the injected current and on the postsynaptic potential at this coupling strength. This is a good example where the closed loop situation of a dynamic clamp experiment leads to partially or incorrectly compensated artifacts altering the overall outcome of the experiments in a highly undesirable way. A further increase in the coupling strength between the two cells results in a sudden magnification in the oscillation amplitude and duration with bridge balance, entirely invalidating the measurement and destroying the experiment at 600 nS synaptic strength. AEC in contrast, could still successfully compensate for the electrode artifact at 1000 nS, where we reached the current injection range limit of our A/D converter (data not shown).

**Fig. 5:**
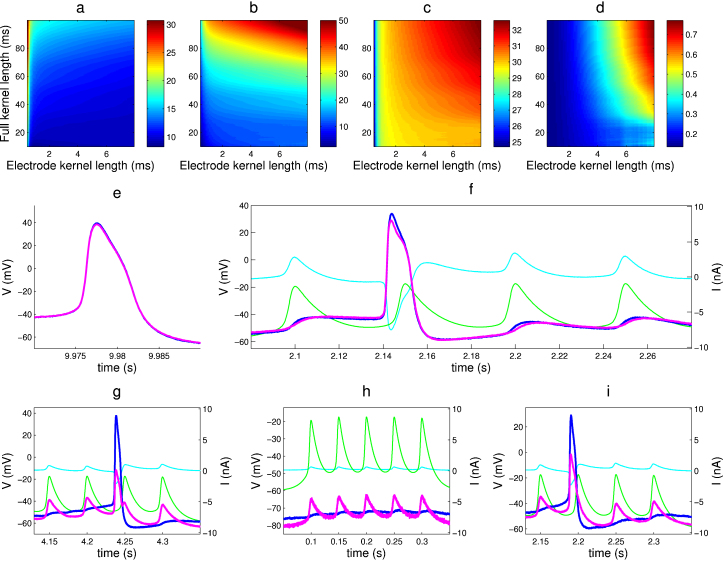
Demonstration of AEC compensation on the Lynmaea stagnalis CGC cell. (a–d) Electrode and (passive) cell membrane properties obtained from the electrode calibration results for a wide region of AEC's two most sensitive parameters (full kernel length and electrode kernel length, see Brette et al. (2008) for details). (e) Spontaneous recorded activity of the cell. (f–i) Compensation results for three investigated electrode artifact compensation techniques, while simulating a symmetric, non-rectifying gap junction synapse between StpdC's spike generator and the CGC: AEC at 100 nS (f), bridge balance at 50 nS (g) and bridge balance and capacitance neutralization combined at 20 nS (h). (i) Example of failed AEC compensation due to a too polarized electrode. Colour code: green: spike generator potential, cyan: injected current, magenta: calculated membrane potential (*V*_raw_ − *V*_*e*_) in case of AEC (f and i), membrane potential as provided by the amplifier in the other cases (e, g and h), blue: control (“true”) membrane potential on an independent electrode and channel.

In the second series of experiments we worked with an identified cell of the mollusc *Lymnaea stagnalis*. The nervous system of the pond snail *Lymnaea stagnalis*, due to its relative simplicity, very short preparation time, and its many, easily accessible, and well characterized neurons, offers an ideal model nervous system for the investigation of a wide range of general neuronal phenomena, from the operation and modulation of central pattern generator circuits, to the neural foundation of associative learning and memory (Kemenes and Benjamin, 2009). In our work to test the different electrode artifact compensation techniques, the size of the *Lymnaea* neurons allowed us to impale them with two electrodes each, and use one electrode to carry out a “single electrode” dynamic clamp experiment, while the other served as an independent, artifact-free control electrode. This setup allows the direct assessment of the compensated signal, if we allow ourselves to neglect the spatial intra-cellular variability of the membrane potential between the two independent electrodes. Although this voltage difference can be significant during the propagation of an action potential through the neuron, depending on the location of the two recording sites, nevertheless, as Fig. 5e illustrates, the two passive electrodes did record the same signal from the cell in our test, with only a small difference on the top of spikes.

We used the CGC cell of *Lymnaea* and performed the same calibration parameter sweep that is presented for the model cell above. Fig. 5a–d shows that when working with the data obtained from this large, tonically spiking cell, we also can identify a broad region within which the calibration procedure results in stable estimates of the electrode properties (*R*_*e*_ and *τ*_*e*_). In fact, the algorithm for StdpC's automatically generated default electrode (*K*_*e*_) and full kernel length (*K*_*f*_) parameters was determined based on these parameter sweep results from the CGC and model cell (see also (Brette et al., 2008) supplementary materials for further theoretical and analytical discussion).

We then conducted a dynamic clamp experiment in which CGC was coupled through a simulated, high-conductance gap junction to StdpC's spike generator. The AEC compensated signal shows very little difference in the shape of the action potential even at very high gap junction coupling strengths (100 nS), with no apparent differences between the actively compensated and the control traces (Fig. 5f, magenta and blue) indicating an almost perfect artifact removal. In comparison, both the bridge balance only and the combined bridge balance and capacitance neutralization configurations show remaining spike-like artifacts on the “compensated” membrane potential signal (magenta traces in Fig. 5g and h) already at lower coupling strengths (50 and 20 nS). This imperfect compensation occurs mainly due to the strong and highly fluctuating currents flowing through the active electrode, and demonstrates the limitations of the traditional artifact compensation techniques for demanding dynamic clamp protocols. Again, as in the case of the model cell (Figs. 1a and 4f), increasing the coupling strength between the pre- and postsynaptic cells led to sudden artifact magnification and high frequency oscillations (at ∼60 nS) when using bridge balance or combined bridge balance and capacitance neutralization, leading to a complete destruction of the experiment. The AEC method on the other hand only showed minor deteriorations in its signal quality even at 200 nS coupling strength (data not shown).

A typical voltage trace from a strongly non-linear electrode is shown in Fig. 5i (magenta trace): the membrane potential is contaminated by a large asymmetric artifact during current injection, indicating that the electrode is heavily polarized (i.e., possesses different conduction characteristics for negative and positive currents, probably due to an abrupt or long term change in the spatial distribution of the ionic concentrations of the electrolyte). Electrodes with this high degree of non-linearity, if identified at the calibration stage, must be replaced. The experimenter is furthermore advised to check the electrode properties from time to time during the experiment (as is the case while using bridge balance compensation), recalibrate any drifted electrodes, and discard non-linear ones.

**Fig. 6:**
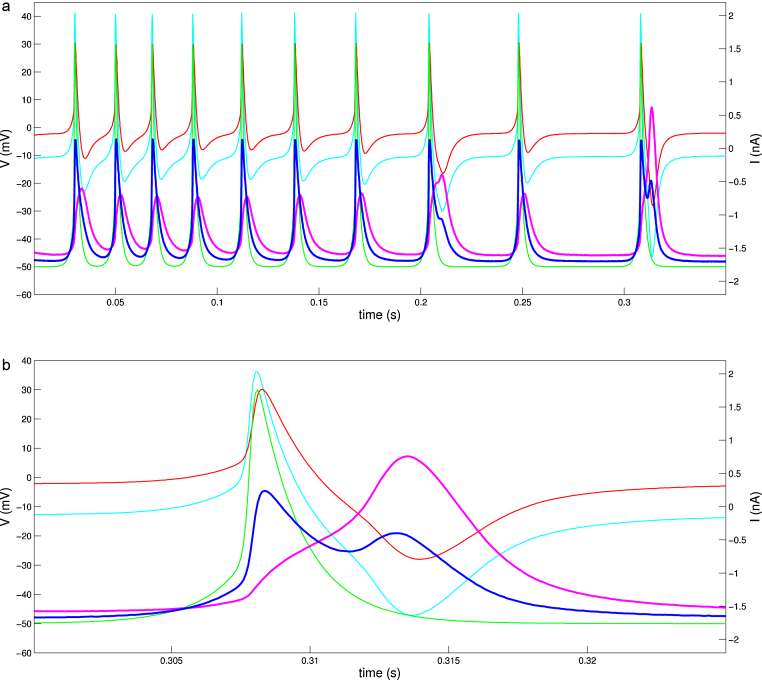
AEC compensation for a patch electrode demonstrated on a cultured rat hippocampal neuron. (a) Repetitive spike generator stimulation of a neuron through a gap junction with 30 nS coupling strength. (b) Last stimulation from a, shown at higher temporal resolution (see time axes). Colour code: green: spike generator potential, cyan: injected current, red: calculated electrode artifact (*V*_*e*_), magenta: calculated membrane potential (*V*_raw_ − *V*_*e*_), blue: raw voltage as provided by the amplifier (uncompensated membrane potential).

In the third set of experiments we investigated the performance of the implemented compensation method for mammalian cells. We applied StdpC's AEC to rat hippocampal neurons interfaced both with sharp electrodes (data not shown) and patch electrodes. This represents a very different electrophysiological configuration than those discussed so far. Fig. 6 shows results obtained from a cultured rat hippocampal neuron which has been patched and connected to the StdpC spike generator with a gap junction synapse of 30 nS maximal conductance. Even though patch electrodes generally have much lower resistances than sharp electrodes, and hence lead to much smaller electrode artifacts, the raw voltage recording from the patch electrode (blue) is still visibly contaminated with an injection artifact. If left uncompensated, this artifact could even be confused with action potentials at positive current injection levels. However, as the higher time resolution Fig. 6b reveals, the cell generated action potential can easily be distinguish by its shape and onset from the spike generator's presynaptic spike (green). Furthermore, from a naïve perspective there is an unexplainable increase in the measured potential during subsequent negative current injections. Taking the calculated voltage drop across the calibrated patch electrode (red) into consideration we recover the qualitatively expected electrode artifact in response to the different current levels, and can reveal the actual cell activity (magenta). Note how the recovered membrane potential shows the expected waveform of a spike and, as there are no signs of the oscillatory artifacts seen during incomplete compensation with other methods, we conclude that AEC was successful in this example as well, even though we do not have a second electrode for direct confirmation on this occasion.

## 6 Discussion

In this paper, we presented a series of experiments to confirm the viability of the active electrode compensation (AEC) method and demonstrate the validity of its implementation in StdpC. First, we started with specially designed test configurations that allowed tight control of the experimental situation, with known resistances and capacitances. This setup does not only allow for (1) the thorough exploration of the calibration procedure's parameter space with respect to the known characteristics of the model cell and (2) the most direct assessment of the compensation accuracy with a fully independent second electrode, but also (3) allowed to test extremely strong coupling (above 600 nS) between the simulated and physical (model) cell, without having to worry about damaging the latter, like in the case of a real neuron. Our results showed that, with respect to the above three points, (1) the calibration process is quite robust with respect to the choice of its two most important parameters (electrode and full kernel length), (2) the AEC compensation shows excellent performance in general, superior to the traditional compensation techniques (bridge balance, and bridge balance with capacitance compensation), and (3) the traditional compensation methods quickly break down with the increase in coupling strength, while our AEC implementation showed respectable accuracy even at the current injection limit of our DAQ board (from +10 to −10 nA).

In the second experimental setup, we utilized the same dynamic clamp protocol of a simulated gap junction between a presynaptic cell simulated within StdpC and the *Lymnaea stagnalis* CGC cell. In order to obtain a direct control measurement of the membrane potential in the same way as in the experiments with the model cell, the neuron was impaled by two electrodes, one for both stimulation and (compensated) recording, and another one for independent control measurement of the membrane potential. The results showed that, although the non-linearities of active membrane processes can interfere with the calibration procedure, it still retains sufficient robustness to be valid even if performed during periods of sparse spiking. The compensation itself again, contrary to the traditional techniques, showed no noticeable flaws in comparison with the control measurement, further validating our implementation in experiments in a challenging dynamic clamp configuration with real neurons.

During our third set of experiments on cultured rat hippocampal neurons, we used the patch clamp technique which has inherently lower resistance in series between membrane and amplifier compared to sharp electrodes. Nonetheless, we could demonstrate the value of using accurate electrode artifact compensation techniques, especially during dynamic clamp experiments, to permit faithful high quality recordings and reveal the true membrane potential response to imposed input. In general, the extreme sensitivity and fragility of mammalian neurons points to the benefit of using such an approach, as it would be very difficult, if not impossible, to work with two electrodes on the same cell. Furthermore, the experiments with the model cell and the *Lymnaea* CGC demonstrated that incomplete electrode artifact compensation as we observed it with conventional methods leads to uncontrolled large errors in the experiments due to the closed loop feedback in dynamic clamp. Any error in artifact compensation results in an error of the calculated injection current for the subsequent dynamic clamp cycle which can quickly escalate to an extent where the experiment becomes meaningless.

As part of the StdpC system, our implementation of AEC operates in a soft-real time environment in which the length of time steps can vary. The general implications and limitations of the soft-real time approach in StdpC have been analyzed previously (Nowotny et al., 2006), demonstrating that measurable distortions of signals, even for fast transients during spiking, are rare. Since this original analysis was published in 2006, the speed of computers has multiplied and, importantly, modern computers all use multi-core CPUs. Distortions due to Windows interrupts have, therefore, practically disappeared. The only limitation that remains in place is that excessive visualisation of signals during dynamic clamp experimentation can still interfere with the main dynamic clamp cycle and hence should be avoided. Taken together, our experimental results show that StdpC, using the AEC feature, is capable of performing challenging dynamic clamp protocols accurately with a single electrode on both robust invertebrate cells recorded intra-cellularly, but also on mammalian cells via patch-recordings. In these validation experiments, only a small fragment of StdpC's dynamic clamp capabilities were used, with the intention to keep the focus on the subject of interest of this paper, namely the fidelity of the implemented electrode compensation technique. Beyond the simulated spike generator and the simple gap-junction model utilized here, StdpC also offers a number of chemical synapse types on up to 6 channels, various spike timing dependent plasticity (STDP) formalisms on any combination of the active channels, a simple but powerful scripting mechanism to gain automated experimentation with precise timing, the ability to save and quickly recover previous setups and dynamic clamp protocols, and a number of other features (see (Nowotny et al., 2006; Kemenes et al., 2011) and StdpC's User Manual).

With the addition of AEC StdpC can now be applied in a majority of dynamic clamp applications that have previously been inaccessible to such experimentation due to the requirements of two independent electrodes. It also allows to reconfirm experiments of authors who have previously used dynamic clamp with single electrodes using only traditional compensation methods of bridge balance and capacitance compensation (Rothman et al., 2009; Rusin et al., 2011).

## Supplementary information

StdpC user manual including detailed troubleshooting instructions.
