# Supplementary material for: Single electrode dynamic clamp with StdpC
Source: J Neurosci Methods. 2012 Oct 15;211(1):11–21. doi: 10.1016/j.jneumeth.2012.08.003 (PMC3482664; doi:10.1016/j.jneumeth.2012.08.003)
Supplement: Supplementary file 1 [file mmc1.pdf]

StdpC 2011

(Spike timing dependent plasticity Clamp),

in parts based on **DYNCLAMP2** [Pinto et al., 2001]

MANUAL

Thomas Nowotny

Centre for Computational Neuroscience and Robotics,
School of Engineering and Informatics,
University of Sussex,
Brighton BN1 9QJ, UK
E-mail: t.nowotny@sussex.ac.uk

Original DYNCLAMP2 developers:

Reynaldo Daniel Pinto,
Robert C. Elson,
Attila Szücs,
M. I. Rabinovich,
A. I. Selverston,
H. D. I. Abarbanel

Institute for Nonlinear Science,
University of California, San Diego,
9500 Gilman Dr.
Mail Code 0402
La Jolla, CA 92093-0402, USA

StdpC is software building on the ideas that led to the development of the earlier DYNCLAMP2 software. The new software is based on QT and has in addition to the original native support for the old DigiData 1200/A interface now also support for all National Instruments devices that support NI's NIDAQmx API.

This is the second beta version of the software. Some functionality has not been tested thoroughly. Any feedback on problems with the software or potential bugs is greatly appreciated. An older version of the software (StdpC) is described in Journal of Neuroscience Methods [Nowotny et al., 2006]. A more recent description of StdpC can be found in Nature Protocols [Kemenes et al., 2011]. *If you use StdpC and publish results based on its use, please cite the website and these papers.* Please contact us for any further questions (t.nowotny@sussex.ac.uk).

Copyright 2011 T. Nowotny

StdpC is free software; you can redistribute it and/or modify it under the terms of the GNU General Public License as published by the Free Software Foundation; either version 2 of the License, or (at your option) any later version.

This program is distributed in the hope that it will be useful, but WITHOUT ANY WARRANTY; without even the implied warranty of MERCHANTABILITY or FITNESS FOR A PARTICULAR PURPOSE. See the GNU General Public License for more details.

You should have received a copy of the GNU General Public License along with this program; if not, write to the Free Software Foundation, Inc., 59 Temple Place - Suite 330, Boston, MA 02111-1307, USA.

1 Introduction

The Dynamic Clamp protocol, developed by [Sharp et al., 1993] and independently by [Robinson and Kawai, 1993] allows inserting simulated membrane conductances into cells, and/or adding synapses between cells as well as a variety of other manipulations. Here, we describe StdpC 2011, a software for controlling biological neurons and an internal spike generator in (soft) real time.

The software allows to define up to 6 artificial synapses, 6 Hodgkin-Huxley conductances [Hodgkin et al., 1949] and one presynaptic artificial neuron (spike generator). The parameters of these simulated entities can be controlled on the graphical user interface (GUI) or through script files.

The StdpC has a built in electrode compensation method (Active Electrode Compensation [Brette et al., 2008], which is optionally usable after going through a short and automatic calibration procedure. This compensation technique allows for application of a single electrode both for stimulation and recording with higher signal fidelity than conventional compensation techniques, like bridge balancing or discontinuous injection/recording.

During the experiment, the acquired data can be saved continuously into a data file either in ASCII or binary format for off-line post-processing and results analysis.

The software supports the DIGIDATA 1200A data acquisition card of Axon Instruments, Inc (now part of MDS Analytical Technologies), all NIDAQmx capable devices of National Instruments, and a pure simulation mode for testing. The program was written in C++ (using the Bloodshed Dev-C++ environment which is based on the popular gcc compiler (mingw)). The graphical user interface is based on Nokia's (formerly Trolltech's) QT libraries. The program has been tested on Windows XP but should also be compatible with earlier versions of Windows (Windows NT, Windows 2000). The actual performance of the dynamic clamp cycle depends on the speed of the PC hardware used. The DIGIDATA driver is based on a modified/extended version of PortTalk by Craig Peacock and is unlikely to work in Windows Vista. The NIDAQmx based driver may work with Windows Vista but has not been tested in this environment.

2 Hardware and software requirements

A standard PC (Pentium IV and up recommended), Windows XP (potentially Windows NT, 2000 ok). A DIGIDATA 1200A ADC/DAC board from Axon Instruments, or a National Instruments DAQ and installed NIDAQmx driver.

The program was tested on a PC with Windows XP professional and a DigiData 1200A board as well as with a NI PCI-MIO-16-4 DAQ using NIDAQmx for Windows, version 8.7.2.

3 Asynchronous Dynamic Clamp Cycle

The dynamic clamp protocol consists of a cycle of reading the membrane potential of the cells, calculating the current to be injected into the cells according to the synapses and conductances that are to be simulated, and generating the voltage commands that will generate the currents. StdpC aims at repeating this cycle at the maximal rate supported by the hardware in order to simulate continuous biological processes. Since Windows is not a real time operating system, the actual time from one cycle to the next can not be controlled in a strict way. Instead of aiming at such a control StdpC is based on an asynchronous dynamic clamp cycle. Every time the program updates the membrane potential of the cells it also reads the real time clock in the Digidata 1200A board (the system clock in case of using NI devices) which allows to calculate the duration of the previous Dynamic Clamp cycle. The measured time intervals between voltage updates are used in the computation of the currents. This limits the impact of unequal delays during repeated cycles unless these delays become large compared to the biological time scales in the system studied. As

a side effect of the asynchronous Dynamic Clamp cycle, StdpC update cycles will always run at the maximum rate supported by the hardware. With the typical successive upgrade of computer hardware the quality of the Dynamic Clamp interaction will improve over time.

4 How to install the software

4.1 Binary package

Download the package and unzip it to a convenient location, e.g., C:\ Program Files\ StdpC. Then execute “StdpC.exe”.

4.2 Source package

Download the source package and unzip it to a convenient location. Open a shell and execute “myqmake.bat”. Then open the dev-c++ project “StdC.dev” and compile. Make sure that the Makefile generated by qmake is used. Note that you will need Dev-C++/mingw and QT 4 installed on your computer. If you are compiling for the NIDAQmx interface you need to have NIDAQmx installed and to enable the option “NIDAQmx” in the “.config” file.

5 Troubleshooting

5.1 I/O conflicts

In the past, there have been problems with I/O conflicts between the DigiData board and other hardware devices. If you are experiencing problems, you may need to change the base address of the DigiData board. Hardware conflicts can occur even if the DIGIDATA board works fine with Axon programs like Axoscope because, unlike these, StdpC 2011 does read the Real Time Counter (RTC) in the Digidata board. Usually, the DigiData board is configured to use the I/O base address 0x320, and in this case the RTC reading ports are in the range of 0x330 - 0x332. Many computers use the address 0x330 for some devices like sound cards, etc.

To check for and resolve a hardware address conflict, *Windows NT, 2000, XP:*
Open: Start → Programs → Administrative Tools → Windows NT Diagnostics. Choose the folder ⟨Resources⟩, click in ⟨I/O Port⟩ and use the cursor to browse the list of used ports. You should look for 330. Note that 320 will not appear because the Digidata Board is not detected by Windows. If you find any reference in the range 330 - 33F and your Digidata board is configured to use the base address 320 you need to change I/O address of your Digidata board to run DynClamp and avoid other problems due to the I/O conflict.
Windows 95, 98:
Open: Start → Settings → Control Panel → System. Choose the folder ⟨Device Manager⟩, click ⟨I/O Port⟩ and use the cursor to browse the list of used ports. You should look for 330. Note that 320 will not appear because the Digidata Board is not detected by Windows. If you find any reference in the range 330 - 33F and your Digidata board is configured to use the base address 320 you need to change the I/O address of your Digidata board to run DynClamp and avoid other problems due to the I/O conflict.
Changing the base address of the Digidata Board and in the StdpC program:
Identify a free range of port addresses. If 0x330 is in use, addresses around 340-370 are usually available which is sufficient for the Digidata ports. Reconfigure the Digidata board for the base address 340 or 350 manually (the RTC will be 350 or 360 and no conflicts!) and run the StdpC program. The Digidata base address can be changed within StdpC on the Settings-¿DAQ dialog.

6 Changes from previous versions/programs

6.1 Changes DYNCLAMP2 to StdpC version 3

The following is a list of features which were new in StdpC v3:

- *Spike generator*
  A spike generator can replace a biological presynaptic neuron. Spikes are generated either periodically in a fixed pattern or from a file containing predefined spike times. This feature is actually an original feature of R. Pinto but was not published yet.
- *Hidden parameter panels*
  To de-clutter the screen of the dynamic clamp computer, in StdpC the parameter dialogs for chemical synapses and Hodgkin-Huxley conductances have been moved to separate pop-up windows.
- *Data displays for debugging*
  To allow for easy debugging of wiring and gain problems the software now has two data displays that can show input and/ or output channels or functions thereof (averages, spikes detected).
- *Save and load parameter settings*
  You can now save and load the current parameter settings of the clamp. This way one does not have to redo parameter settings every time the StdpC program is restarted. The settings are stored in a simple ASCII format that allows editing by hand or snooping around for curiosity.
- *Spike timing dependent plasticity*
  The two chemical synapses can now be made plastic obeying a Spike Timing Dependent Plasticity protocol. There are two different protocols to choose from and a variety of parameters determining the details of the learning mechanism
- *Experimental automatization and scripting*
  The StdpC supports a simple form of experimental protocol automation (scripting). The user can specify a script file that contains events at given times. These events include switching on and off of synaptic connections or Hodgkin-Huxley type conductances as well as arbitrary parameter changes. The script is loaded on starting the clamping process and executes the commands at the given times after clamping started.

6.2 Changes from StdpC version 3 to version 4

- Since this version of StdpC, all synapses are freely reconfigurable as chemical or electrical synapses.
- the presynaptic and postsynaptic neuron of each synapse is freely reconfigurable. In particular it is now possible to combine synapses between two biological neurons (on channels IN0/OUT0 and IN1/OUT1) with synapses from the spike generator (channel SPG) to either of the neurons. This gives more flexibility in the experimental design
- Each chemical synapse can now been chosen individually to be plastic or not.
- The initial value for a plastic synapse is given by its individual G_s setting, not by a global initialisation value in the plasticity block. The displays for the synaptic strength have been removed because they turned out to be disruptive for the clamping performance.
- Some smaller adjustments aiming at better user interaction and performance of the data displays. We, however, still recommend not to use the data displays during real experiments. They are for debugging purposes only and degrade clamping performance considerably when used.

6.3 Changes from StdpC v4 to StdpC 2007

- The user interface is now based on QT
- The driver for the DigiData 1200 board was re-created using (an extended version of) the free PortTalk package.
- StdpC 2007 supports all NIDAQmx capable National Instruments boards
- The source code has been re-organised to allow for rapid inclusion of additional hardware support.
- StdpC 2007 allows to use all input and output channels available on the acquisition hardware.
- All synaptic properties including the details of the synaptic plasticity are now individually adjustable for every synapse
- There are now two alternative descriptions for Hodgkin-Huxley type conductances, including several functional forms for the activation and inactivation curves.
- With the additional support for other hardware, there is now more fine-grained control over the channels that are used for voltage and current conversion. All channels are available in "input channels" and "output channels" dialogs. Only channels activated in these dialogs will appear in the drop-down menus within synapses and HH conductances.

6.4 Changes from StdpC 2007 to StdpC 2010

- Active Electrode Compensation feature included as an optional, automatic, digital electrode compensation technique calculated within the software.
- A new feature allows for the measurement of the resistance and capacitance properties of both the electrode and cell membrane, conveniently controlled from the software.
- Full experimental data saving support has been included. Any combination of the active input and output channels, along with the spike generator, can be saved, with a frequency (quasi) independent of the main dynamic clamp loop frequency. Data can be saved either in ASCII, or in binary format.
- A constant bias current can now be defined for any active output channel for special experiment configuration needs.

6.5 Changes from StdpC 2010 to StdpC 2011

- Active electrode compensation is now stable.
- Through scripting, a sample-and-hold mechanism can be envoked if measurement artefacts due to the experimental protocol need to be removed from voltage signals. For example, the monitoring of membrane potentials can be interrupted during stimulation from sources external to the dynamic clamp.
- The synapse models have been updated with the addition of the "Destexhe synapse" and a modification of the alph-beta synapse.
- A variety of minor bugs and issues have been fixed.
- Data saving can now be enabled and disabled globally in teh data saving dialog. This overrides the choices for individual channels, and if disabled, the overheads of this mechanism can be avoided.

7 How to use StdpC

7.1 "Connecting" the program to the cells

To avoid the generation of wrong current commands due to artifacts introduced into the membrane measurement by the current injection, one must either (1) insert of two electrodes into each cell (one for injection, the other one for recording), or (2) use of a careful compensation of a single electrode in order to recover the actual membrane potential from the raw artefactual voltage. For the latter solution StdpC has a built-in artifact compensation method, AEC (Active Electrode Compensation [Brette et al., 2008]), that allows for a convenient, software-based electrode compensation. One can thought of special experiment configurations, like simultaneously recoding in multiple machines, in which AEC might not be found appropriate for compensation. In these cases any external analogue technique the microelectrode amplifier features could be applied instead of (and NOT in conjunction with) AEC. However we generally encourage the application of AEC, as it is an automatic method that provides high quality signals at arbitrary high time resolution. See section 9 for more detail about electrode compensation.

The membrane potential output of the microelectrode amplifiers should be connected to the analog inputs channels chosen to be scanned in the "input channels" dialog. The command voltage for the injection of current into the cells will be in the analog output channels chosen in the corresponding drop-down boxes. For specific channels to appear in these boxes, they need to be activated in the "output channels" dialog. The built-in spike generator is internally mapped to be input channel SG.

The currents are calculated individually for each conductance and synapse and then are summed for each cell to generate the corresponding voltage command for the microelectrode amplifiers. It is recommended to turn on the dynamic clamp cycle and to monitor the current command voltage in an oscilloscope before turning on the injection of the current in the microelectrode amplifier, to see if it looks like what is expected. If it is orders of magnitude wrong, check the conversion factors in the "input channels" and "output channels" dialogs. Alternatively, the channel setup settings can be checked and corrected safely on a model cell, which are usually shipped with the microelectrode amplifiers.

7.2 Control Panel of the Program

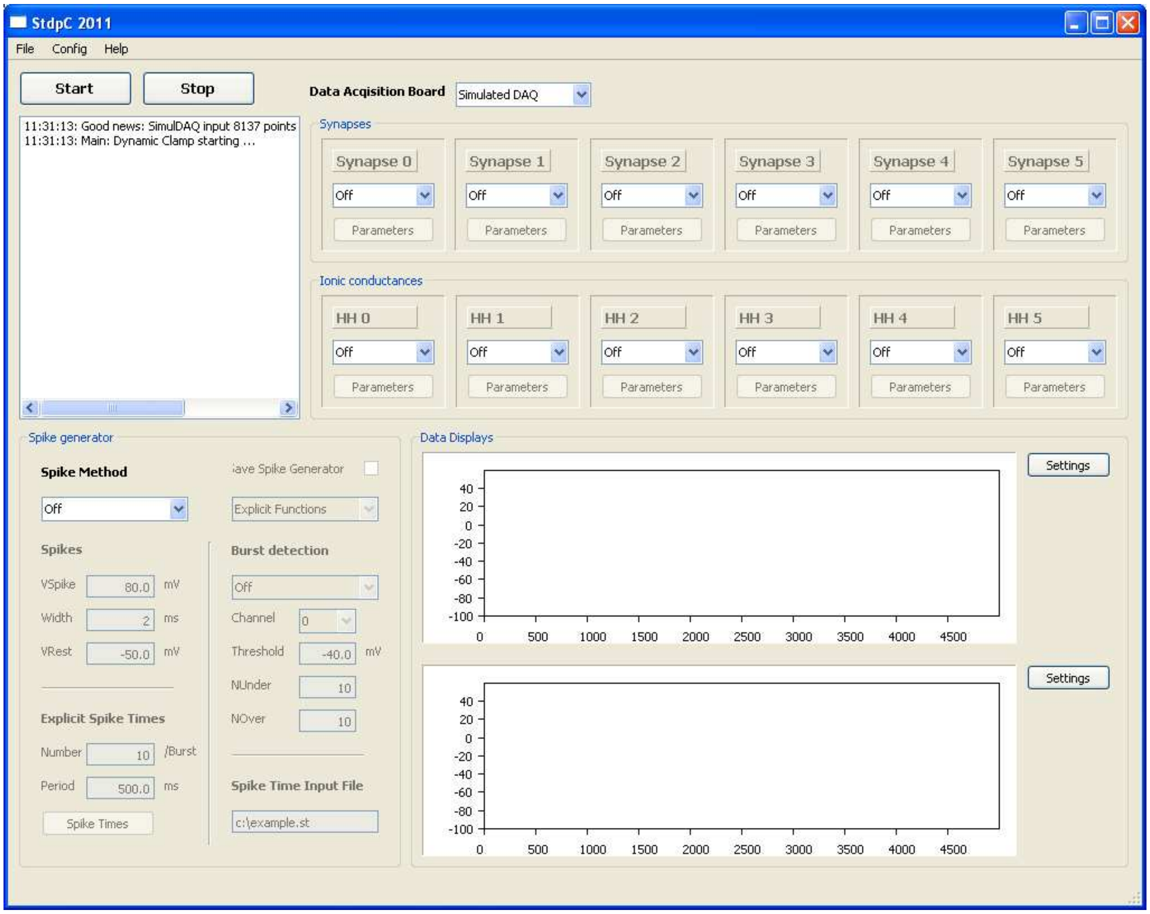

The main control panel contains different blocks of control for the different functions of the software – simulating synapses (synapse control block; 1), simulating ionic conductances (Hodgkin-Huxley (HH) type conductances; HH control block; 2), simulating presynaptic activity (spike generator block; 3) and displaying data (data displays; for debugging only; 4). The control blocks are front-ends to more detailed control dialogs. There is a message window in the upper left part of the panel that shows system messages. These messages can be saved for future reference. They are also automatically written to a local file "StdpC_last.log".

7.3 Configuration of the Hardware and Control of the Program

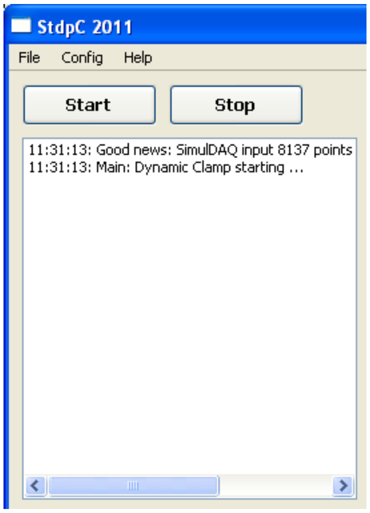

Once StdpC is started, the last known hardware configuration is loaded (from a local file named "StdpC.conf"). Other parameters are initialized with standard values that are pre-compiled into the software. The message window will show a message whether the chosen hardware was successfully initialized (which was not the case in the example shown on the left).
Failing hardware initialization can have several causes:

1. If using the DigiData 1200(A):
   StdpCs default address for the board is 0x320 (hexadecimal), which is the default address of the board, but the specific board can have been configured to use another range of addresses. Possible values are 0x340, 0x360, 0x380, 0x3A0, 0x3C0, etc. Make sure that the correct address is entered in the "DAQ" dialog.
2. If using a National Instruments board:
   Make sure that NIDAQmx is installed and the board is working properly (using the NI Automation explorer). Make sure your device is in the list of devices that support NIDAQmx (as opposed to the older devices supporting "old style" NIDAQ / NIDAQ legacy only.
3. I you are running with simulated data acquisition:
   Make sure that you have a correctly formatted input file for the membrane potential of cells and that the file name in the "DAQ" dialog points to the right location of this file. Also make sure that the location the output file name points to is writable to you.

The remaining controls are the "start" and the "stop" button. The "start" button initiates the start of dynamic clamp cycles. In particular, pressing this button will stop a running dynamic clamp thread, load the settings from (almost) all dialogs into memory, and start the dynamic clamp thread again with the new settings. The dynamic clamp thread will then run until stopped by pressing the "stop" button.

The data acquisition hardware can be chosen in the combo box on the top. Note that the software that has been compiled with support for the NIDAQmx libraries will not run if these are not installed *even if a different device is chosen*. The specific hardware settings can be adjusted in the "DAQ" dialog int the "Config" menu of the main menu bar.

The choice of hardware, its settings and the settings for input and output channels are the only exception to the rule that changes only apply after a restart of the dynamic clamp thread (by pressing the "start" button).

Other general control elements can be found in the menus of the main menu bar.

- "File" menu:
  - "Load Protocol": Load parameter settings from a previous session. The standard file name extension for these is "cpr", albeit the settings are saved in plain ASCII format.
  - "Save Protocol": Save the current parameter settings into a file for later use or documentation.
  - "Load Script": Load a script for experiment automation. Scripting is described in section 8.
  - "Unload Script": Remove a script from memory and use StdpC interactively.

- "Export Log": Export the contents of the message box to a file.
- "Clear Log": Remove the message box contents.
- "Exit": Quit StdpC.

- "Config" menu:
  - "DAQ": Controls for the properties of the data acquisition hardware. This will show a dialog window that is specific to the chosen hardware.
  - "Input Channels": Brings up a dialog to change settings for the analog input channels, see below for more details.
  - "Output channels": Brings up a corresponding dialog for the output channels, see below.
  - "Electrode compensation"Shows Electrode Compensation dialog for electrode calibration, see below.
  - "Data saving" Shows Data Saving dialog to configure data saving settings, see below.

- "Help": Offers a one-line description about StdpC.

7.4 Electrical synapses

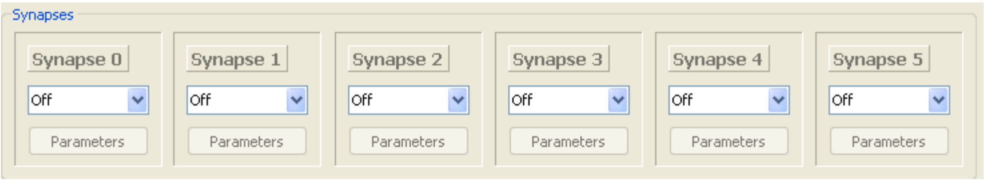

Each subunit in the synapses block can be configured to be a chemical or an electrical synapse (gap junction). Clicking the "Parameters" button will open a control panel that allows to change properties of the synapse.

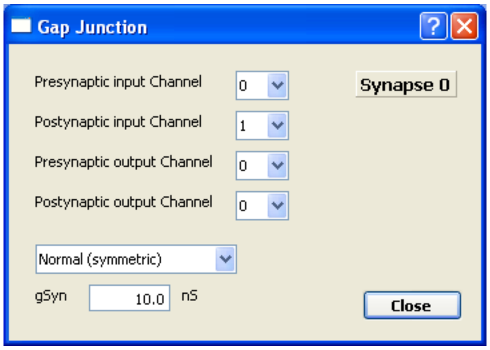

If configured as an electrical synapse the control window on the left will appear.
The currents I_{pre} and I_{post} to be injected into the pre- and post-synaptic cells, respectively, are calculated according to:

$$I_{\text{post}}(t) = g_{\text{Syn}}[V_{\text{pre}}(t) - V_{\text{post}}(t)], \quad (1)$$

$$\text{and} \quad I_{\text{pre}}(t) = -I_{\text{post}}(t), \quad (2)$$

where $V_{\text{pre}}(t)$ and $V_{\text{post}}(t)$ are the membrane potential of the two cells. If the gap junction is chosen as rectifying, $I_x(t) = 0$ if $V_{\text{pre}}(t) < V_{\text{post}}(t)$.

The control elements are

- Presynaptic input channel: Combo to choose the input channel that contains the membrane potential of the presynaptic cell (SP= spike generator)
- Postsynaptic input channel: Combo to choose the input channel that contains the membrane potential of the postsynaptic cell
- Presynaptic output channel: Combo to choose the output channel that will contain the current command for the presynaptic cell.

- Postsynaptic output channel: Combo to choose the output channel for the current command intended for the postsynaptic cell.
- Type combo: Choose whether the gap junction is ordinary (the current can flow from the presynaptic cell to the postsynaptic cell an vice versa; both cells have perfectly symmetrical roles in this case) or rectifying (positive current can only from the presynaptic cell to the postsynaptic cell but not in the other direction).
- gSyn: Conductance of the gap junction in nS.

7.5 Chemical Synapses

If the type of synapse is chemical, the control dialog of a chemical synapse is displayed on pressing "Parameters". The current to be injected into the postsynaptic cell, I_{post}, is calculated in each dynamic clamp cycle using a first order kinetics model of the release of neurotransmitter and an additional inactivation term, $h(t)$, to simulate short term depression:

$$I_{\text{post}} = g_{\text{Syn}}\, S(t)\, h(t)[V_{\text{Syn}} - V_{\text{post}}(t)], \tag{3}$$

where the instantaneous activation, S(t), and inactivation, h(t), terms are given by the differential equations

$$(1 - S_\infty(V_{\text{pre}}))\tau_{\text{Syn}} \frac{dS(t)}{dt} = (S_\infty(V_{\text{pre}}) - S(t)) \tag{4}$$

$$\tau_h \frac{dh(t)}{dt} = h_\infty(V_{\text{pre}}) - h(t), \tag{5}$$

where

$$S_\infty(V_{\text{pre}}) = \begin{cases} \tanh\left[\frac{V_x(t) - V_{\text{Thresh}}}{V_{\text{Slope}}}\right] & \text{if } V_{\text{pre}} > V_{\text{Thresh}} \\ 0 & \text{otherwise} \end{cases} \tag{6}$$

$$h_\infty(V_{\text{pre}}) = \frac{A}{1 + \exp\left(\frac{V_{\text{pre}} - V_{\text{Thresh}}}{V_{\text{Slope}}}\right)}, \tag{7}$$

$$\tau_h(V_{\text{pre}}) = \tau_0 - \frac{A_\tau}{1 + \exp\left(\frac{V_{\text{pre}} - V_{\text{Thresh},\tau}}{V_{\text{Slope},\tau}}\right)} \tag{8}$$

The controls in the parameter dialog for the chemical synapse are

- Presynaptic Channel: Combo box to choose the input channel that contains the membrane potential of the presynaptic cell (SP = spike generator)
- Postsynaptic Channel: Combo box that contains the membrane potential of the postsynaptic cell
- Output Channel: The output channel to which the current command for the synapse will be added

"Standard":

- gSyn: The maximal conductance g_{syn} of the synapse in nS.
- VSyn: The reversal potential V_{Syn} in mV.
- tauSyn: The characteristic time constant τ_{Syn} of the synapse in ms.

- VThresh: The threshold potential V_{Thresh} for the release of neurotransmitter in mV.
- VSlope: The "slope" parameter V_{Slope} of the activation curve in mV.

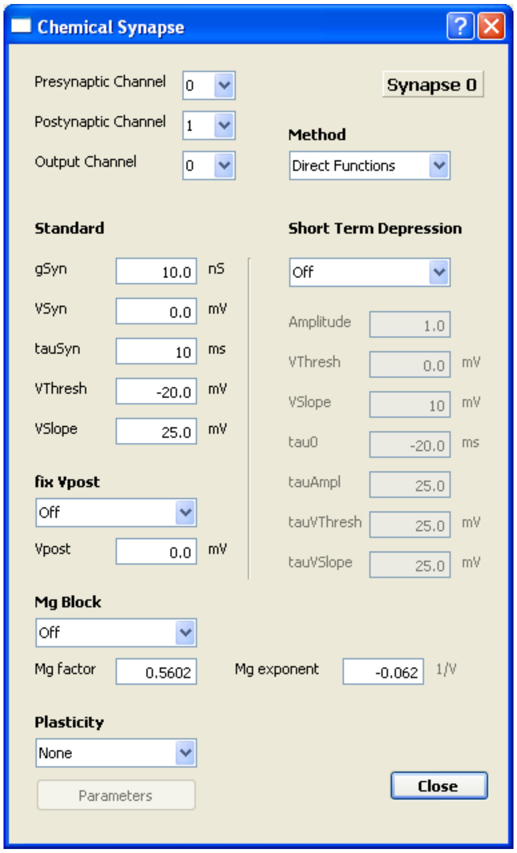

"Short Term Depression":

- Combo box to switch this mechanism on or off
- Amplitude: The amplitude of the $h(t)$ depression variable. Typically set to 1 (so that h varies between 0 and 1). This was previously used to switch short term depression on or off and remains for historical reasons.
- VThresh: The threshold potential $V_{\mathrm{Thresh},\tau}$ for the activation of h in mV.
- VSlope: The "slope" parameter of the depression activation curve in mV.
- tau0, tauAmpl, tauVThresh, and tauVSlope: Parameters τ_0, A_τ, $V_{\mathrm{Thresh},\tau}$ and $V_{\mathrm{Slope},\tau}$ for the voltage-dependent characteristic time τ_h of short term depression.
- The plasticity combo box allows to choose whether the synapse is subject to long term plasticity and according to which model the plasticity is determined.

For "Spike STDP" a spike-timing based rule is applied according to the parameters defined in the corresponding control panel that appears upon clicking the "Parameters" button. If "ODE STDP" is chosen, the synaptic plasticity is implemented according to the ordinary differential equation (ODE) description in [Abarbanel et al., 2002]. Parameters again are adjusted in the separate panel that appears after pressing "Parameters".

7.6 Alpha beta synapse

The $\alpha\beta$ synapse implemented in StdpC is a variation on the classic Rall synapse [Rall, 1967] with a pre-synaptic release variable R and a post-synaptic binding variable S which then gates the synaptic current. The model is described by:

$$I_{\mathrm{syn}} = g_{\mathrm{syn}} S (V_{\mathrm{rev}} - V_{\mathrm{post}}) \tag{9}$$

$$\frac{dS}{dt} = \alpha_S (1 - S) R - \beta_S S \tag{10}$$

$$\frac{dR}{dt} = \alpha_R (1 - R) \frac{1}{1 + \exp(\frac{V_{\mathrm{pre}} - V_{\alpha R}}{s_{\alpha R}})} - \beta_R R \tag{11}$$

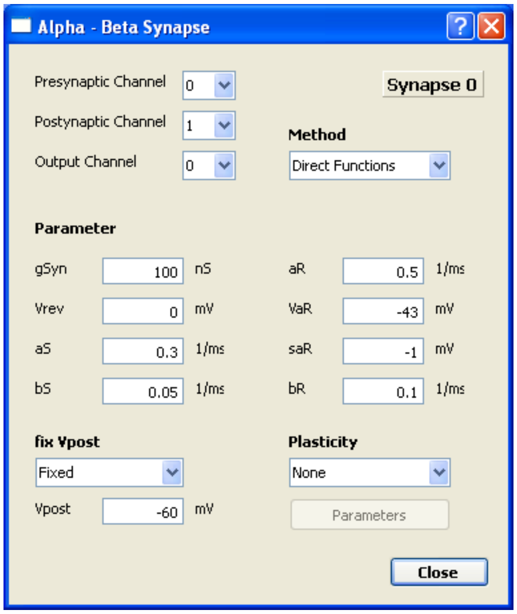

- Presynaptic Channel, Postsynaptic Channel and Output Channel are as in other synapse models
- The method for evaluating the sigmoid function is chosen in the Method combo. Unless speed problems are encountered "Direct Functions" is recommended.
- gSyn: Maximal synaptic conductance
- Vrev: Reversal potential of the synapse
- aS: Rate of transmitter binding to post-synaptic targets
- bS': Rate of transmitter removal (channel closing) postsynaptically
- aR: Rate of transmitter release when pre-synaptic potential is elevated
- VaR: Midpoint of the sigmoid function expressing the level of pre-synaptic release as a function of pre-synaptic membrane potential
- saR: Width of the release sigmoid function
- bR: Fall rate of the presynaptic release
- The remaining parameters, including plasticity are as in the other synapse models.

7.7 Destexhe synapse

his synapse model is the simple first order synapse description introduced in [Destexhe et al., 1994]. In brief,

$$I_{\text{syn}} = g_{\text{syn}} S(V_{\text{rev}} - V_{\text{post}}) \tag{12}$$

$$\frac{dS}{dt} = \begin{cases} \alpha(1-S) - \beta S & \text{if } t - t_{\text{spike}} < t_{\text{release}} \\ -\beta S & \text{otherwise} \end{cases} \tag{13}$$

where t_{spike} is the time of the occurence of the last spike in the pre-synaptic neuron.

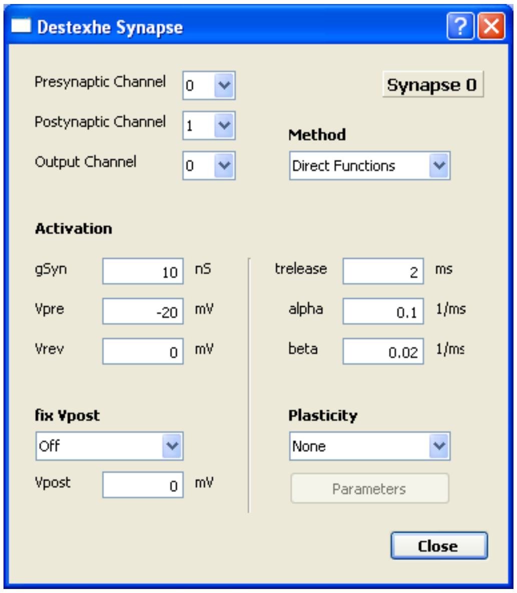

- Presynaptic Channel, Postsynaptic Channel and Output Channel are as in other synapse models
- The method for evaluating the sigmoid function is chosen in the Method combo. Unless speed problems are encountered "Direct Functions" is recommended.
- gSyn: Maximal synaptic conductance
- Vpre: pre-synaptic threshold for triggering synaptic transmitter release
- Vrev: Reversal potential of the synapse
- trelease: The time (duration) of transmitter release after a presynaptic spike
- alpha: The rise rate for the EPSCs
- beta: the fall (decay) rate for the EPSCs
- The remaining parameters, including plasticity are as in the other synapse models.

7.8 Spike Timing Dependent Plasticity

As described above, synapses can be equipped with a form of Spike Timing Dependent Plasticity (STDP). The typical way of implementation, denoted as "Spike STDP", is to detect spikes in the pre- and postsynaptic cells and define

$$\Delta g = \pm A_{\pm} \left(\frac{|\Delta t - \tau_{\text{Shift}}|}{\tau_{\pm}} \right)^{q} \exp\left(- \frac{|\Delta t - \tau_{\text{Shift}}|}{\tau_{\pm}} \right). \tag{14}$$

Δg is then added to the "raw" synaptic conductance g_{raw} whenever a pre- or postsynaptic spike occurs. Note that for correct function, spike detection needs to be switched on for the pre- and postsynaptic input channels (see input channel dialog). The synaptic conductance g_{Syn} is then determined from g_{raw} through a sigmoid filter

$$g = g_{\max} \tanh\left(\frac{g_{raw} - g_{\text{Mid}}}{g_{\text{Slope}}} \right) \tag{15}$$

to avoid problems of "run-away" potentiation.

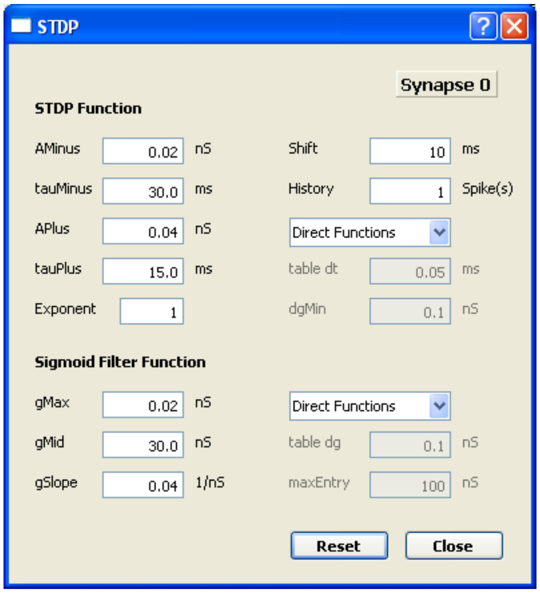

The control parameters are:
"STDP Function"

- AMinus: The amplitude A_- of the negative (left) part of the STDP curve.
- tauMinus: The time scale τ_- (locus of the extremum) of the negative (left) part of the STDP curve.
- APlus: The amplitude A_+ of the positive (right) part of the STDP curve.
- tauPlus: The time scale τ_+ (locus of the extremum) of the positive (right) part of the STDP curve.
- Exponent: The exponent q of the polynomial factor in the STDP curve.
- Shift: The offset τ_{Shift} of the STDP curve on the Δt axis.
- History: The number of spikes to be considered in the other neuron if a spike occurs in a given neuron. For example, if set to 1 and a spike occurs in the postsynaptic neuron, the change Δg will only be calculated and applied for the last spike that occurred in the presynaptic neuron. If it was 2 it would be calculated for the last and the next to last spike in the presynaptic neuron.
- The method combo box allows to choose whether the STDP function is calculated directly with the appropriate C functions when needed or whether it is tabled up front and this lookup-table is being used.
- table dt: The time step in the lookup table.
- dgMin: The minimum value for Δg that should be in the table. This determines how far to the left and right the table covers the STDP curve.

"Sigmoid Filter Function"

- gMax: The maximal g_{Syn} allowed.
- gMid: The midpoint g_{Mid} of the filter.
- gSlope: The "slope" parameter g_{slope} of the sigmoid filter.
- The method combo allowing the choice between direct calculation or lookup tables for the filter function.
- table dg: The stepping in terms of g_{raw} of the lookup table.
- maxEntry: The maximal g_{raw} entry in the table.

Alternatively the synaptic strength is governed by a set of differential equations according to the model of synaptic plasticity in [Abarbanel et al., 2002]. In this case, the maximal synaptic

conductance g_{Syn} is subject to a differential equation system

$$\frac{dP}{dt} = v_{\text{pre}} - \beta_P P \tag{16}$$

$$\frac{dD}{dt} = v_{\text{post}} - \beta_D D \tag{17}$$

$$\frac{dg_{\text{raw}}}{dt} = \gamma(PD^\eta - DP^\eta). \tag{18}$$

The normalized voltages v_x are derived from the measured potentials V_x through capped linear filters

$$v_{\text{pre}} = \begin{cases} 0 & V_{\text{pre}} < V_{P,\text{min}} \\ \frac{V_{\text{pre}} - V_{P,\text{min}}}{V_{P,\text{max}} - V_{P,\text{min}}} & V_{P,\text{min}} \leq V_{\text{pre}} \leq V_{P,\text{max}} \\ 1 & V_{P,\text{max}} < V_{\text{pre}} \end{cases} \tag{19}$$

and accordingly for v_{post},

$$v_{\text{post}} = \begin{cases} 0 & V_{\text{post}} < V_{D,\text{min}} \\ \frac{V_{\text{post}} - V_{D,\text{min}}}{V_{D,\text{max}} - V_{D,\text{min}}} & V_{D,\text{min}} \leq V_{\text{post}} \leq V_{D,\text{max}} \\ 1 & V_{D,\text{max}} < V_{\text{post}} \end{cases} \tag{20}$$

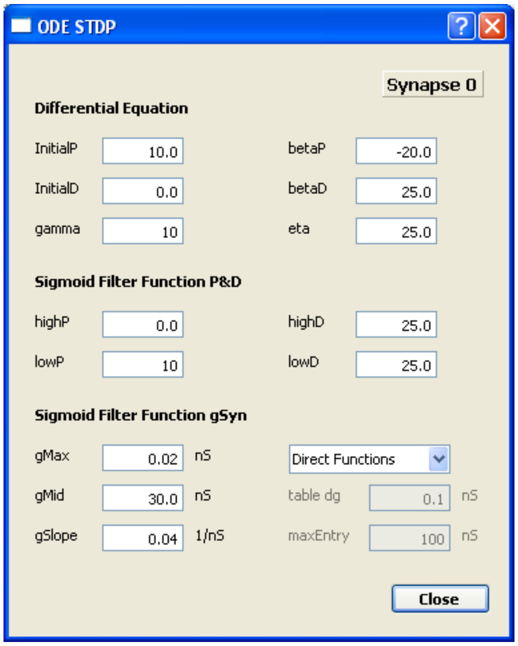

The control parameters for ODE based STDP are: "Differential Equation"

- InitialP: Initial value for the "potentiation variable" P
- InitialD: Initial value for the "depression variable" D
- gamma: Exponent γ
- betaP: Rate of decay β_P of P in 1/ms= kHz.
- betaD: Rate of decay β_D of D in 1/ms= kHz.
- eta: Rate of change of g_{raw} in nS/ms.

"Sigmoid Filter Function P & D"

- highP: The upper limit $V_{P,\text{max}}$ of the P filter
- lowP: The lower limit $V_{P,\text{min}}$ of the P filter
- highD: The upper limit $V_{D,\text{max}}$ of the D filter
- lowD: The lower limit $V_{D,\text{min}}$ of the D filter

"Sigmoid Filter Function gSyn"

- gMax: The maximal allowed value for g_{Syn}.
- gMid: The mid point of the sigmoid filter for g_{Syn}
- gSlope: The "slope" parameter of the sigmoid filter function for g_{Syn}.

- The method combo allows to switch between direct evaluation of the filter or the use of a lookup table
- table dg: The step size in the lookup table
- maxEntry: The maximum of g_{raw} for which table entries are generated.

7.9 Hodgkin-Huxley Type Conductances

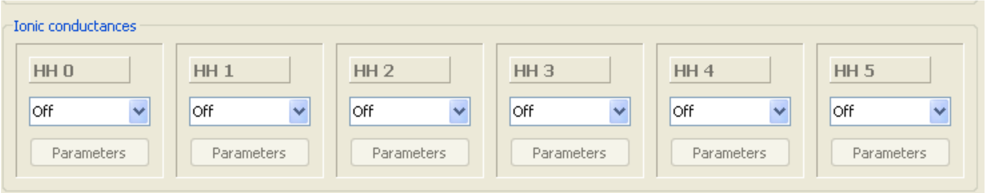

StdpC can inject up to six Hodgkin-Huxley type ionic conductances into cells. The six conductances can be inserted into a single cell or into several cells in any desired combination. The control blocks of Hodgkin- Huxley type conductances allow to choose two different types for the description.

For the "m/h/tau" description, the current I_{HH} to be injected into a cell with membrane potential V(t) is calculated according to

$$I_{HH}(t) = g_{\text{Max}} m(t)^p h(t)^q (V_{\text{rev}} - V(t)), \tag{21}$$

where m and h are modelled as

$$\tau_m \frac{dm}{dt} = m_\infty(V) - m, \tag{22}$$

$$\tau_h \frac{dh}{dt} = h_\infty(V) - h, \tag{23}$$

with steady state values

$$m_\infty = \frac{1}{1 + \exp\left(\frac{V - V_m}{s_m}\right)}, \tag{24}$$

$$h_\infty = \frac{1}{1 + \exp\left(\frac{V - V_h}{s_h}\right)}, \tag{25}$$

and time scales

$$\tau_m = \tau_{0,m} - \frac{A_{\tau,m}}{1 + \exp\left(\frac{V - V_{\tau,m}}{s_{\tau,m}}\right)}, \tag{26}$$

$$\tau_h = \tau_{0,h} - \frac{A_{\tau,h}}{1 + \exp\left(\frac{V - V_{\tau,h}}{s_{\tau,h}}\right)}, \tag{27}$$

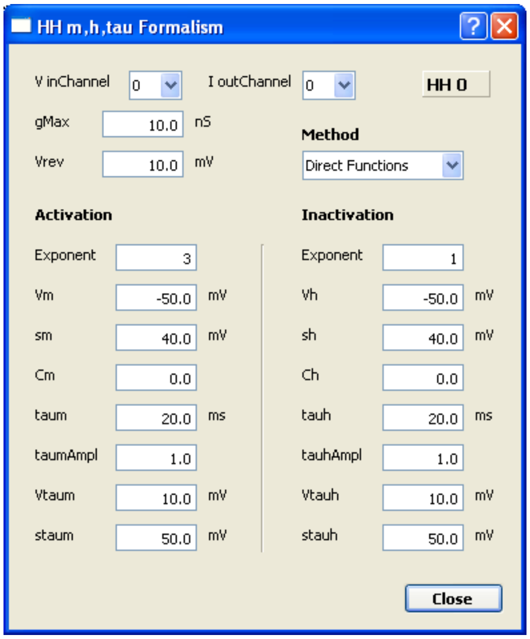

Control parameters of the ionic conductances in the m/h/tau formalism are:

- V in Channel: The input channel that contains the membrane potential of the cell the conductance is injected into
- I out Channel: The output channel to which the current command for the inserted ionic conductance will be added
- gMax: The maximal conductance g_{Max} of the inserted channel
- Vrev: The reversal potential V_{rev} of the inserted channel
- Method: The combo box allows to choose between direct evaluation of C functions or a pre-calculated lookup table.

"Activation"

- Exponent: The exponent p in the current equation
- Vm: The activation potential V_m
- sm: The width s_m of the activation function
- Cm: The offset parameter C_m for persistent currents
- taum: The minimal time scale $\tau_{0,m}$ for τ_m.
- taumAmpl: The range (Amplitude) $A_{\tau\, m}$ for the time scale τ_m.
- Vtaum: The mid point $V_{\tau,m}$ for the time scale sigmoid function.
- staum: The "slope" parameter $s_{\tau,m}$ for the time scale sigmoid function

"Inactivation"

- Exponent: The exponent q in the current equation
- Vh: The inactivation potential V_h
- sh: The width s_h of the inactivation function
- Ch: The offset parameter C_h for persistently inactivated currents
- tauh: The minimal time scale $\tau_{0,h}$ for τ_h.
- tauhAmpl: The range (Amplitude) $A_{\tau,h}$ for the time scale τ_h.
- Vtauh: The mid point $V_{\tau,h}$ for the time scale sigmoid function.
- stauh: The "slope" parameter $s_{\tau,h}$ for the time scale sigmoid function.

Alternatively, the ionic conductances can be described in a α/β formalism, where

$$I_{\mathrm{Syn}} = g_{\mathrm{Max}} m^p h^q (V - V_{\mathrm{rev}}) \tag{28}$$

$$\frac{dm}{dt} = \alpha_m(1-m) - \beta m \tag{29}$$

$$\alpha_m = k_{\alpha,m} F_{\alpha,m}\left(\frac{V - V_{\alpha,n}}{s_{\alpha,m}}\right) \tag{30}$$

$$\beta_m = k_{\beta,m} F_{\beta,m}\left(\frac{V - V_{\beta,n}}{s_{\beta,m}}\right) \tag{31}$$

$$\frac{dh}{dt} = \alpha_h(1-h) - \beta_h h \tag{32}$$

$$\alpha_h = k_{\alpha,h} F_{\alpha,h}\left(\frac{V - V_{\alpha,n}}{s_{\alpha,h}}\right) \tag{33}$$

$$\beta_h = k_{\beta,h} F_{\beta,h}\left(\frac{V - V_{\beta,n}}{s_{\beta,h}}\right). \tag{34}$$

The “activation”/”inactivation” functions $F_{\bullet,\bullet}$ can be chosen from three choices,

$$F_1(x) = \frac{x}{\exp(x) - 1} \tag{35}$$

$$F_2(x) = \exp(x) \tag{36}$$

$$F_3(x) = \frac{1}{1 + \exp(x)}. \tag{37}$$

Note, that this formalism allows to implement, for example, the classic neuron model by Traub and Miles [Traub and Miles, 1991].

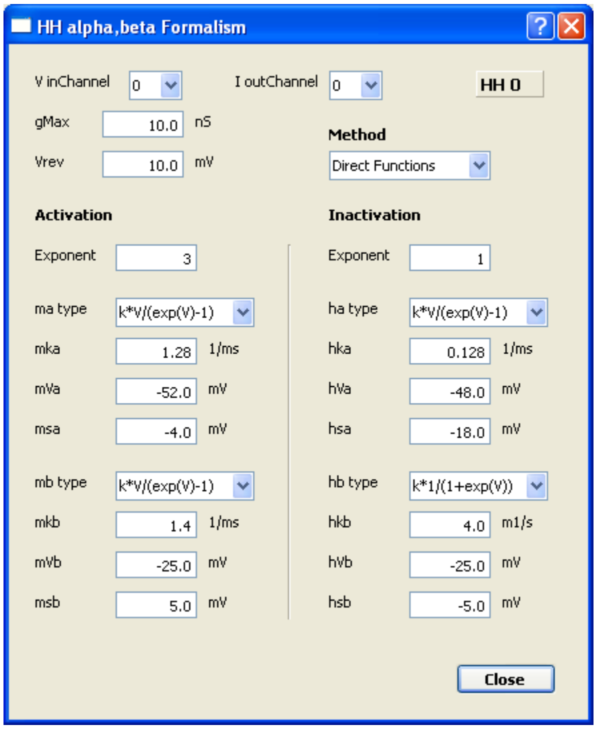

The control parameters are

- V in Channel: The input channel that contains the membrane potential of the cell the conductance is injected into
- I out Channel: The output channel to which the current command for the inserted ionic conductance will be added
- gMax: The maximal conductance g_{Max} of the inserted channel
- Vrev: The reversal potential V_{rev} of the inserted channel
- Method: The combo box allows to choose between direct evaluation of C functions or a pre-calculated lookup table.

"Activation"

- Exponent: The exponent p in the current equation
- ma type: The choice of the functional form for $F_{\alpha,m}$.
- mka: Rate parameter $k_{\alpha,m}$ for the rise of m
- mVa: The midpoint $V_{\alpha,m}$ for the sigmoid function for α_m
- msa: The "slope" parameter $s_{\alpha,m}$ for the sigmoid function for α_m
- mb type: The choice of the functional form for $F_{\beta,m}$.
- mkb: Rate parameter $k_{\beta,m}$ for the decay of m
- mVb: The midpoint $V_{\beta,m}$ for the sigmoid function for β_m
- msb: The "slope" parameter $s_{\beta,m}$ for the sigmoid function for β_m

"Inactivation"

- Exponent: The exponent q in the current equation
- ha type: The choice of the functional form for $F_{\alpha,h}$.
- hka: Rate parameter $k_{\alpha,h}$ for the rise of h
- hVa: The midpoint $V_{\alpha,h}$ for the sigmoid function for α_h
- hsa: The "slope" parameter $s_{\alpha,h}$ for the sigmoid function for α_h
- hb type: The choice of the functional form for $F_{\beta,h}$.
- hkb: Rate parameter $k_{\beta,h}$ for the decay of h
- hVb: The midpoint $V_{\beta,h}$ for the sigmoid function for β_h
- hsb: The "slope" parameter $s_{\beta,h}$ for the sigmoid function for β_h

7.10 Spike generator

The spike generator unit can replace a presynaptic cell. It can operate in several very different modes. When its input are spike timings, the spikes have a pre-defined form. Alternatively it can replay a voltage waveform from a file directly. The spike time information can either be explicit spike times from a file, spike patterns (bursts) specified in a file, or a spike pattern defined explicitly through the graphical interface. The pattern options can be combined with burst detection, in which each spike pattern is triggered by a detected threshold event in another (measured) neuron.

The control elements for the spike generator are

- The "Spike Method" combo allows to choose the type of operating method – explicit spike times from the GUI, spike times from a file (that includes explicit times if burst detection is not enabled, or burst patterns if it is), and replay of a voltage waveform from a file.
- The "Save Spike Generator" checkbox allows the user to choose if the spike generator channel would be saved or not (only if the spike generator is on in any of its possible modes)
- The method combo allows to choose for the use of functions or lookup tables.

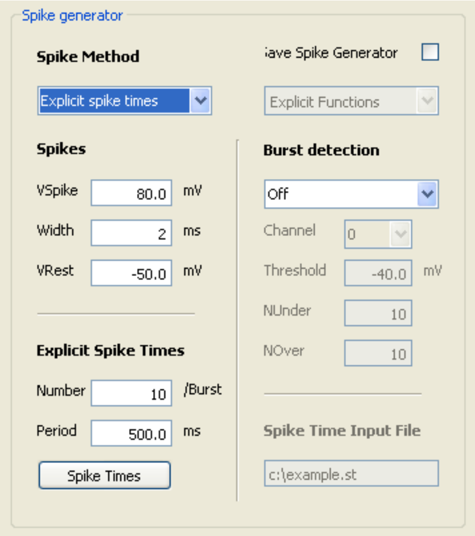

"Spikes"

- VSpike: The amplitude of the spikes generated
- Width: The width of spikes in ms
- VRest: Th resting potential from which the spikes depart

"Explicit Spike Times"
If this method was chosen, the parameters are

- Number: The number of spikes in the pattern (burst)
- Period: The period with which to repeat the spike pattern periodically
- The "Spike Times" button will make the window visible, in which explicit spike times can be entered

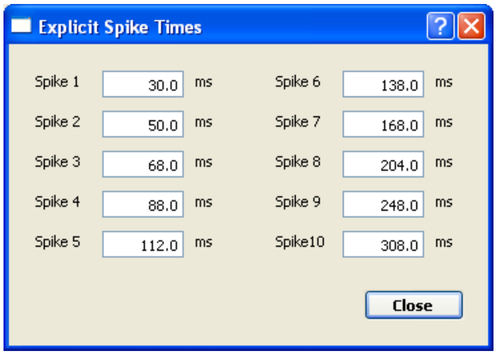

"Burst detection"

- The transition combo allows to choose whether to trigger an event for low to high or high to low transitions through a threshold value
- Channel: The input channel on which to detect events
- Threshold: The trigger threshold for event detection. For low→high detection, an event is triggered whenever NUnder measurements were below threshold and afterwards NOver measurements were above. Note that for noisy signals it may be necessary to increase both numbers for reliable detection. The events below and above are not required to be contiguous in the current implementation.
- NUnder: see "Threshold" above.
- NOver: see "Threshold" above.

"Spike Time Input File"

- The input file name for the file that contains the spike time information. If burst detection is off, the file should contain one clear text column containing times in seconds when spikes shall occur. If burst detection is on, StdpC expects descriptions of spike patterns containing of
  - An integer denoting the number of spikes in the pattern
  - A matching number of spike times in seconds, measured from the detection event.

  These pattern descriptions are best separated by newlines.

7.11 Data displays

The two data displays in StdpC are very useful to check the parameter settings chosen. Typical errors in dynamic clamp setups are wrong conversion factors on input- and output channels. By displaying the data acquired on input channels in the graph displays, some of these errors can easily be detected and corrected.

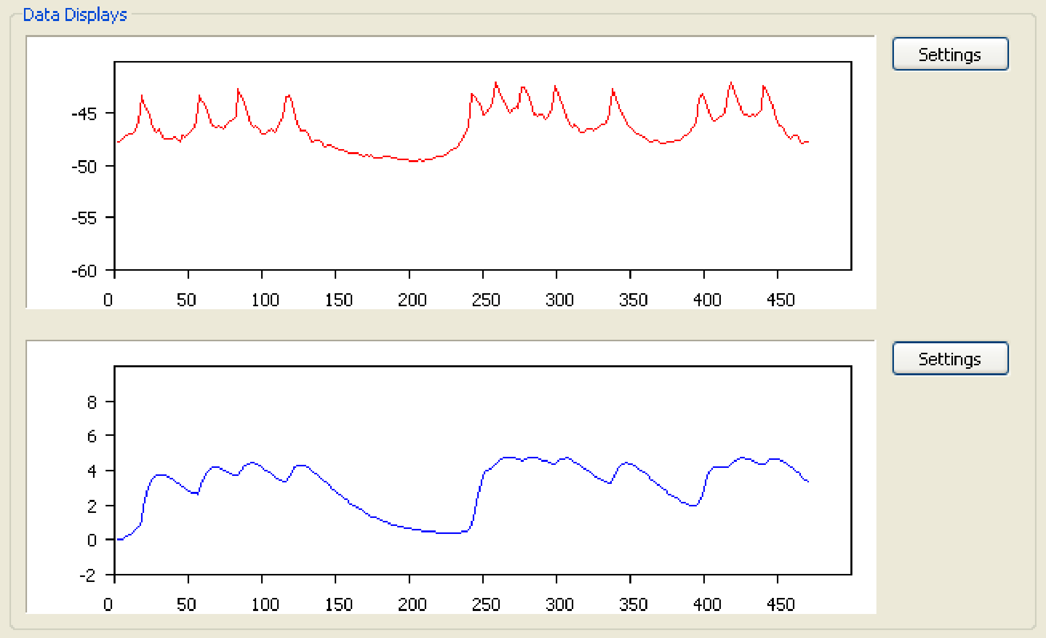

The graph panels are controlled by the dialog that appears when pressing “Settings”.

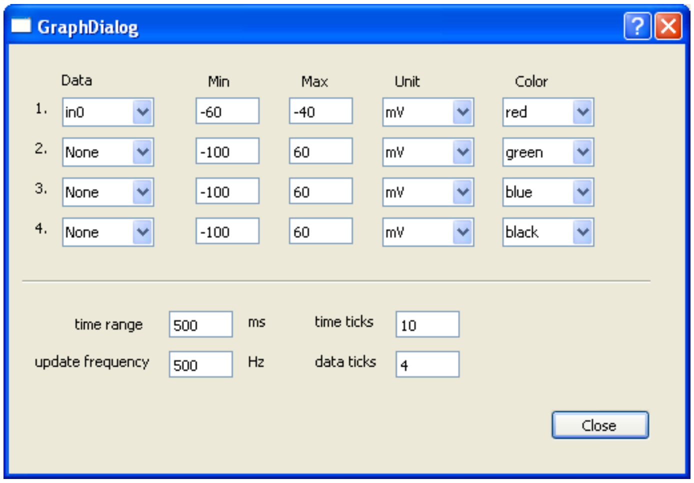

The control elements are:

- Data column: Combo boxes to choose which data to display
- Min: Minimum on the y axis for this data
- Max: Maximum ion the y axis for this data
- Unit: Units in which Min and Max are expressed
- Color: Choice of color of the data trace.

- time range: The length of the x axis in ms
- time ticks: Number of ticks on the x axis.
- update frequency: The rate with which points on the display are updated. Note that a too high rate will likely lead to an overload and subsequent freeze of the program. A more efficient and safe implementation of the displays is on the todo list.

8 Experiment automation

So far we have described how to control StdpC using the graphical user interface. Instead, or in addition, most parameters can be controlled through a scripting mechanism. Through the File → Load Script one can specify a script file. The script file should contain three columns:

1. A time in seconds when a certain event shall occur
2. A parameter name in clear text which shall be changed
3. A new value for the parameter in question

The value column can contain double precision numbers or integers depending on the parameter being changed.

The script file will be read into a list of events in memory such that changes to the file have no effect after the script was loaded.

The parameter names are the same as used when the state of the GUI is saved ("save protocol"). However, not all parameters can reasonably changed while the clamp is running. A list of the most common parameters one may want to change through scripting are given in the table.

Please note that internally, all parameters and variables are in SI units V, A, s, S, etc. The same applies to values in the script files. The variable i in the variable names in the table has to be replaced by the appropriate number of the synapse or conductance, $i \in \{0, \ldots, 5\}$.

9 Electrode compensation

For the compensation of electrode artifacts, StdpC uses the so-called Active Electrode Compensation (AEC) algorithm, developed by Romain Brette and his colleagues. Here we only discuss those parts of this cutting-edge compensation technique, that are necessary for the StdpC end-user to be aware of, however we also encourage to user to consult [Brette et al., 2008] and its supplementary material for the full description of the algorithm.

The AEC method consist of two parts: an initial calibration phase and the subsequent compensation phase happening during the actual experiment. The first part is the calibration phase, during which StdpC first injects a probing current into the cell (which is an uncorrelated white noise signal), and records the voltage response of the system (comprising of the cell and the electrode) to that current. Based on the acquired signal-response relationship, the software then calculates the actual parameter values of the used electrode for the general AEC model. Having had the electrode calibrated, in the second phase, StdpC automatically uses this digital model of the electrode on the corresponding input channel, and calculates the artefactual voltage drop across the electrode occurring on the recording of that channel due to current injection through the electrode.

9.1 Basic considerations and troubleshooting for electrode compensation

High fidelity artifact compensation requires well-calibrated electrodes, thus the calibration phase is a crucial part of the electrode compensation process. Even though it is implemented in the most automated way possible, there are several points the user must make sure before calibration and take care after calibration (just like in the case of any other calibration technique). Briefly these are the following (see [Brette et al., 2008] Supp. Mat. for details):

- *Do the calibration only when the electrode is already in the cell.* Electrode properties change after cell membrane impalement, and the electrode has to be calibrated in its stable state.
- *Make sure that the electrode is (sufficiently) linear in terms of its steady state resistance.* One of the basic assumptions of AEC is that the steady state electrode voltage response changes linear

Name	value range
CSynp[i].ST.AMinus	double
CSynp[i].ST.tauMinus	double
CSynp[i].ST.APlus	double
CSynp[i].ST.tauPlus	double
CSynp[i].ST.Exponent	int
CSynp[i].ST.Shift	double
CSynp[i].ST.History	int
CSynp[i].ST.tableDt	double
CSynp[i].ST.tableDgMin	double
CSynp[i].ST.gMax	double
CSynp[i].ST.gMid	double
CSynp[i].ST.gSlope	double
CSynp[i].ODE.InitialP	double
CSynp[i].ODE.InitialD	double
CSynp[i].ODE.betaP	double
CSynp[i].ODE.betaD	double
CSynp[i].ODE.gamma	double
CSynp[i].ODE.eta	int
CSynp[i].ODE.highP	double
CSynp[i].ODE.lowP	double
CSynp[i].ODE.highD	double
CSynp[i].ODE.lowD	double
CSynp[i].ODE.gMax	double
CSynp[i].ODE.gMid	double
CSynp[i].ODE.gSlope	double
CSynp[i].gSyn	double
CSynp[i].VSyn	double
CSynp[i].tauSyn	double
CSynp[i].VThresh	double
CSynp[i].VSlope	double
CSynp[i].STD	0-1
CSynp[i].STDAmpl	double
CSynp[i].STDVThresh	double
CSynp[i].STDVSlope	double
CSynp[i].STDtauAmpl	double
CSynp[i].STDtau0	double
CSynp[i].STDtauVThresh	double
CSynp[i].STDtauVSlope	double
CSynp[i].fixVpost	0-1
CSynp[i].Vpost	double
CSynp[i].Plasticity	0-2
ESynp[i].type	0-1
ESynp[i].gSyn	double
mhHHp[i].gMax	double
mhHHp[i].Vrev	double
mhHHp[i].mExpo	int
mhHHp[i].hExpo	int
mhHHp[i].Vm	double
mhHHp[i].sm	double

Name	value range
mhHHp[i].Cm	double
mhHHp[i].taum	double
mhHHp[i].taumAmpl	double
mhHHp[i].Vtaum	double
mhHHp[i].staum	double
mhHHp[i].Vh	double
mhHHp[i].sh	double
mhHHp[i].Ch	double
mhHHp[i].tauh	double
mhHHp[i].tauhAmpl	double
mhHHp[i].Vtauh	double
mhHHp[i].stauh	double
abHHp[i].gMax	double
abHHp[i].Vrev	double
abHHp[i].mExpo	int
abHHp[i].hExpo	int
abHHp[i].maFunc	0-2
abHHp[i].mka	double
abHHp[i].mVa	double
abHHp[i].msa	double
abHHp[i].mbFunc	double
abHHp[i].mkb	double
abHHp[i].mVb	double
abHHp[i].msb	double
abHHp[i].haFunc	0-2
abHHp[i].hka	double
abHHp[i].hVa	double
abHHp[i].hsa	double
abHHp[i].hbFunc	0-2
abHHp[i].hkb	double
abHHp[i].hVb	double
abHHp[i].hsb	double
SGp.VSpike	double
SGp.spkTimeScaling	double
SGp.VRest	double
SGp.bdThresh	double
SGp.bdNUnder	double
SGp.bdNOver	double
SGp.period	double
SGp.SpikeNo	int
SGp.SpikeT[0]	double
SGp.SpikeT[1]	double
SGp.SpikeT[2]	double
SGp.SpikeT[3]	double
SGp.SpikeT[4]	double
SGp.SpikeT[5]	double
SGp.SpikeT[6]	double
SGp.SpikeT[7]	double
SGp.SpikeT[8]	double
SGp.SpikeT[9]	double

with the amplitude of the current level injected into it. The Electrode Compensation dialog has a built-in feature to assess the linearity of the electrode (see below). If you experience too high deviation in the steady-state voltage levels in response to different voltage levels (e.g. above 10% of the electrode resistance), we recommend to change the electrode, and rerun the linearity test for the new one.

- *Make sure that the electrode has reached an equilibrium state in terms of its steady state resistance before calibration.* During experiment, any change in the electrode properties after calibration would result in an error in the artifact compensation. The most varying property is the resistance of the electrode, which can change remarkably especially in the first few minutes after cell impalement. Thus one must always make sure that the steady-state electrode resistance is not changing any more before calibration. The electrode measurement feature on the Electrode Compensation dialog provides a convenient way for this assessment (see below).
- *Make sure that the electrode is at least ten times faster than the cell membrane.* Another basic assumption of the AEC calibration phase is that the electrode and the passive cell membrane (ie. not spiking, but subthreshold) responses are separable in the time domain. This usually satisfied in general electrode/cell configurations, but does not stand in case of a very slow electrode and a very fast cell membrane. The Electrode Calibration dialog provides means for the measurement of both electrode and cell membrane response properties (resistance levels, time constants and their standard deviations, see below). As in the case of large cells it can be difficult to effectively drive and measure the cell's subthreshold properties, generally one can satisfy this criterion by making sure that the electrode time constant (which can be measure more reliably) is around half millisecond or less. If the electrode has been found too slow, one can use a certain level of capacitance neutralization (CN) during the calibration and the compensation. This CN level must be set before calibration, and left unchanged during the usage of the same calibrated model of the electrode. See also next point for applying CN.
- *Turn other compensation techniques off before calibration and during compensation as well.* AEC is a complete electrode compensation method. The simultaneous operation of any other active electrode compensation technique, like bridge balancing or discontinuous injection/recoding, would lead to disastrous compensation results. The only exception from this rule is the capacitance neutralization feature of many microelectrode amplifier, which is able to increase the artifact compensation fidelity of AEC, if it is carefully set to the maximum possible level before calibration and left unchanged during the experiment (see previous point).
- *Assess actual data acquisition frequency after calibration.* Right after calibration the user should make sure that StdpC had been able to run the data acquisition part of the compensation in a relatively constant frequency by observing the *Std in sampling rate* text field in the *Data acquisition results* subpanel (in the bottom right corner). The software automatically warns the user if this deviation is higher than 0.01, what results in a degradation in the quality of the electrode calibration and thus in the subsequent artifact compensation as well. Most of the time the popping up of this warning means that a significant operation system interruption has occurred during calibration (it is also possible that the hardware (PC and/or DAQ) is not able to reach the required sampling rate set in the top right corner, in which case lowering the rate should solve the issue). To avoid the operation system interruption problem, and also to reach the best clamping quality, we strongly recommend to stop all computational intensive processes the computer might run in the background during calibration and dynamic clamping.
- *Recalibrate if electrode properties has changed over time.* As for all electrode artifact compensation technique, the compensation quality degrades if changes in the electrode properties (mostly in the resistance) have occured as a long experiment goes on. To rule out this possibility, we recommend the check of the electrode resistance or even the recalibration of the electrode from time

to time during the experiment, and also the assessment of the electrode after the experiment has finished to validate the quality of the compensation.

9.2 Supported features and the dialog panel

Along with the electrode calibration, the Electrode Compensation dialog has two auxiliary features to facilitate calibration and subsequent artifact compensation at high quality.

The *electrode measurement* feature acquires the electrode resistance and time constants according to a simple parallel RC circuit model by injecting different constant current levels into the electrode and simultaneously recording the onset-dynamic and equilibrium level of the voltage response. There are three important things to assess here: (1) the electrode is fast enough (ie. that its average time constant is less then about 1 ms), (2) the electrode is linear enough (ie. that the standard deviation in its resistance levels at different current levels is below 10% of its absolute average resistance), and (3) the electrode has reached an equilibrium level in its resistance (ie. the average electrode resistance does not change remarkably in between subsequent measurements). Also see the troubleshooting subsection above for a more detailed discussion of these issues. It is crucial to find a long enough, but not too long injection length while the electrode is in the cell, in order to let the electrode reach its steady state voltage response, but in the same also time prevent the cell membrane to markedly influence the measurement of the electrode characteristics. A rule of thumb is to set the injection length between 3 and 5 times of the expected electrode time constant, what usually leads to injection lengths around 1 and 2 ms. An incidental spike of the cell simultaneously with the electrode measurement always result in incorrectly measured properties, this case the one should remeasure the electrode.

The *cell membrane measurement* feature allows for the measure of the same (passive) properties of the cell membrane, based on the same parallel RC model of the membrane and the already acquired electrode characteristics. This measurement is done by injecting a particular current step into the cell, holding it for a certain duration, then stepping back to zero current level, waiting for the same amount of time to allow the membrane potential to recover, and then doing the same injection/waiting cycle for a user-settable number of times. This feature is included to allow the user to get a rough idea about the time constant of the cell membrane and this way the appropriateness of the electrode for AEC (the electrode must be at least 10 times faster than the passive cell membrane for good quality artifact compensation) Also see the troubleshooting subsection above for a more detailed discussion of this issues. Please be aware, that because of the nature of measurement protocol, it is likely to provide good cell properties, only if (1) the electrode has been measured previously and its resistance has not changed significantly meanwhile, (2) the passive membrane response is at least one order of magnitude slower than the electrode (only required for precise membrane time constant calculation), and most importantly (3) the injected current is able to effectively drive the membrane potential of the cell, that is, no indenpendent activity of the cell (let it be sub- or suprathreshold) intervene and contaminate the measurement remarkably. This last point implies that successful membrane property measurement of large cells probably requires high amplitude currents, if it is possible at all with this technique. As large cells usually have slower membrane, in these cases the user can satify the "time-separability" criterion (see Troubleshooting section) by making sure that they do not use a very slow electrode (ie. time constant is lower than 1ms).

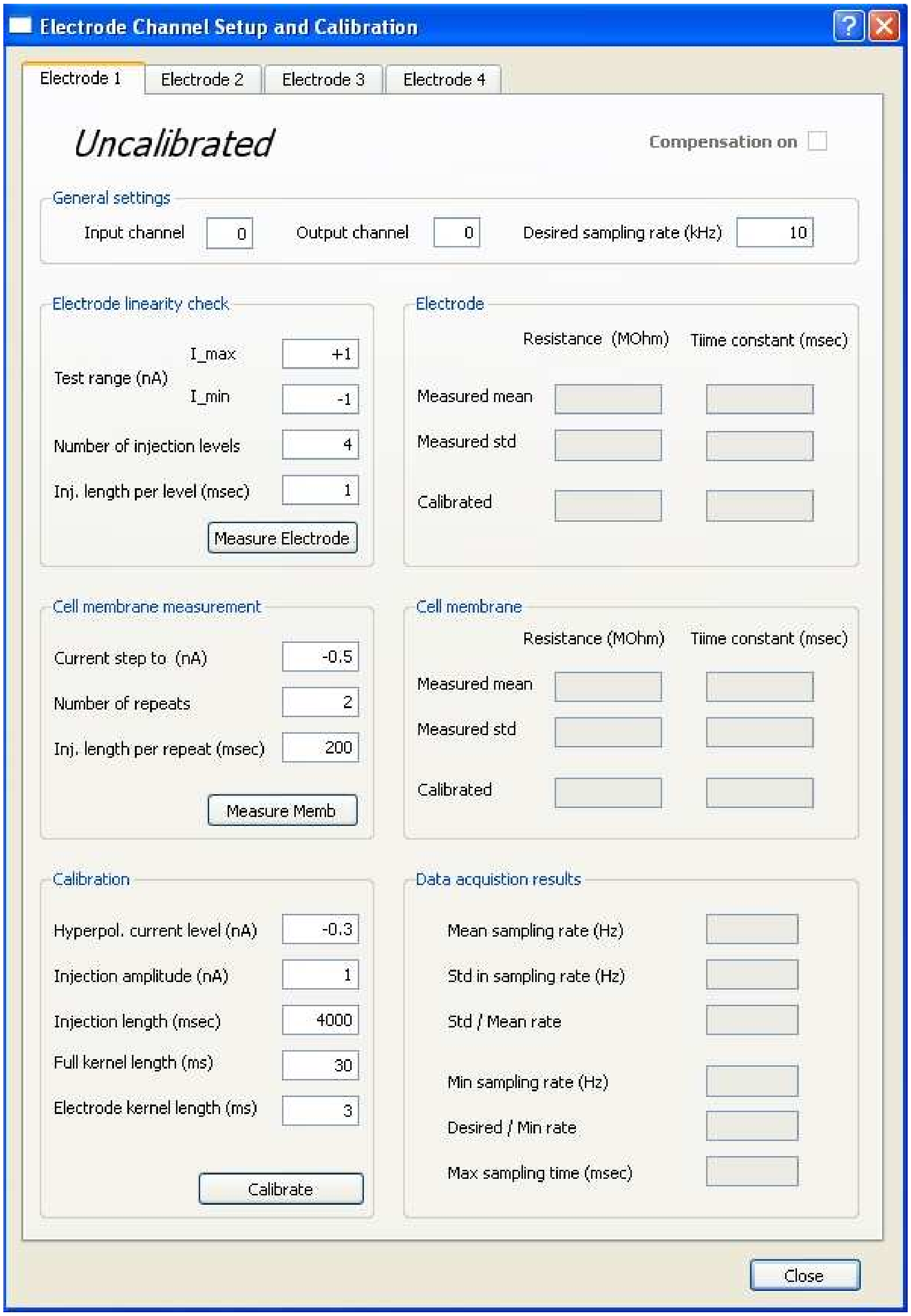

The dialog allows for the calibration of four electrodes (Electrode 1 to Electrode 4) through the tabular menus on the top of the calibration panel. The control and result elements on each tab page are the following:

- Information line: Each page has a text line on the top (saying *Uncalibrated* on the included picture) to provide some basic information about the calibration process for the user.
- *Compensation on*: after calibration, this checkbox allows the user to turn the compensation on

and off on the corresponding electrode.

- *General settings*: This panel contains the settings that applies to all the available features (ie. the electrode measurement, the membrane measurement and the calibration) on the current tabular page.
- *Input channel*: The number of the input channel assigned to the electrode, must be one of the active input channels.
- *Output channel*: The number of the output channel assigned to the electrode, must be one of the active output channels.
- *Desired sampling rate*: The sampling frequency that StdpC will aim to keep during the calibration or the electrode/membrane measurement.
- *Electrode linearity check*: This panel contains the settings specific for the electrode measurement feature. See also *General settings*.
- *I_max*: Maximal current level injected into the electrode.
- *I_min*: Minimal current level injected into the electrode.
- *Number of injection levels*: Number of current levels injected during electrode measurement. The current levels are equally spaced between the maximal and minimal levels, with the limits included. For example, if I_max = 1 nA, I_min = -1 nA and *Number of current levels* = 4, then the current levels to be injected are $I_1 = -1nA, I_2 = -1/3nA, I_3 = +1/3nA, and I_4 = +1nA$. Zero current level is always skipped.
- *Inj. length per level*: defines how long the injection of each current level lasts.
- *Cell membrane measurement*: This panel contains the settings specific for the cell membrane measurement feature. See also *General settings*.
- *Current step to*: Sets the current step at which the cell properties will be measured. Negative current steps are highly recommended, as depolarization increases the chance that active membrane phenomena, like spiking, will disrupt the measurement of passive membrane properties.
- *Number of repeats*: defines how many time the above current level will be injected into the cell. Higher repeat numbers give numerical stability to the measurement, but only if no spike has occured during any of the injections.
- *Inj. length per repeat*: defines how long each injection will lasts. Also defines the recovery time in between current level injections.
- *Calibration*: This panel contains the settings specific for the electrode electrode calibration feature. See also *General settings*.
- *Hyperpol. current level*: allows for the injection of a certain constant level of hyperpolarization current into the cell during the whole duration of the calibration in order to minimize the number of spikes occurring during calibration. High hyperpolarization levels (ie. above -1nA) are not favourable, as they can remarkably change the electrode characteristics.
- *Injection amplitude*: the amplitude of the injected white noise current signal. Setting 1 nA here usually means a good tradeoff between reachin a high signal-to-noise ratio and avoiding driving the cell into an active state (like spiking) in the same time. Always make sure, that the DAQ hardware allows for the issuing of this current level (both to positive and negative levels).

- *Injection length*: defines how long the current injection during the calibration phase lasts. In most cases, a 4-5 second long calibration is sufficient to acquire statistically significant amount of data for the electrode parameter calculation.
- *Full kernel length*: one of the crucial parameters of the AEC technique. A rule of thumb is to set it to one or two times of the time constant of the membrane. After cell membrane measurement StdpC automatically fills this field, however, as the membrane property measurement can be quite imprecise in some cases (huge cell, changing electrode properties or spiking activity), always check that it is somewhere in between 30 and 60 ms before issuing the calibration command.
- *Electrode kernel length*: the other very important parameter for the calibration. A rule of thumb here is to set it to 10 times of the time constant of the electrode, but to no more than 5ms. After electrode measurement StdpC automatically fills this field, however, always check that it is somewhere in between 1 and 5 ms before issuing the calibration command.

10 Saving of experiment data

A new feature in StdpC is the experiment data saving facility. The user can choose between binary of ASCII data formats, pick any subset of the active channels (see Section 11 and 12) including the voltage of spike generator as an input channel, set the data saving frequency and also find a file which the experiment data will be saved into. After starting the dynamic clamping, in each clamping cycle the software decides whether it is time to perform a saving by compering the last saving time and the current time. This way the saving frequency is quasi independent of the dynamic clamping frequency. When a data saving occurs, a new line is being added to the data file obeying the following pattern:

$$Time\_stamp \quad Input\_chnl\_1 \quad ... \quad Input\_chnl\_n \quad Output\_chnl\_1 \quad ... \quad Output\_chnl\_n$$

where the spike generator is always the last input channel (*Input_channel_n*), if it has been set to be saved.

Please be aware, that, as no offline compression is implemented in the current version, StdpC can generate huge data files during long experiments. The size of the saved data can be reduced by

- choosing binary format rather than ASCII
- decreasing the saving frequency
- only saving the channels which are going to be important for the data post-processing and analysis

For a simple comparison, the simulation of a gap junction (electrical synapse) between two neurons produces about 30 MByte/minute data if the user wants to save all the four channels (2 input and 2 output channels + the extra time stamp) at 10kHz frequency in ASCII mode, while the same experiment produces about 3.6 MByte/minute data in case the user only saves the membrane voltages of the two cells (2 input channels + the extra time stamp) at 5kHz in binary mode. Having finished the experiment, the user can load the experiment data file into any data analysis platform they prefer (Matlab, R, Octave, Scilab, etc) for the offline processing and analysis of the data.

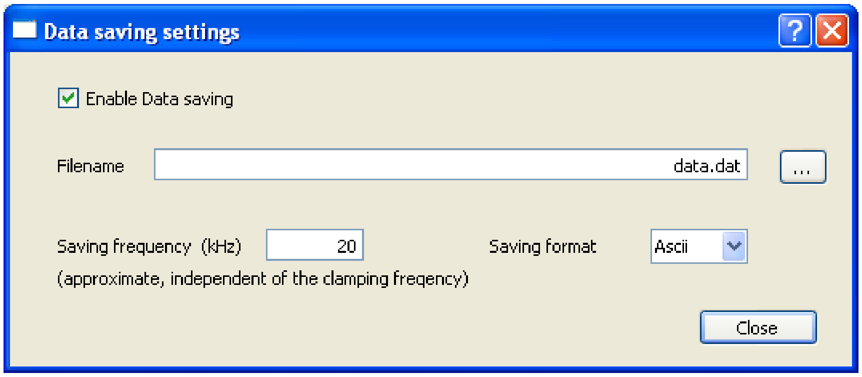

The configuration settings on the *Data saving settings* dialog are the following:

- Filename: the text line indicates which file is selected for data saving. The user must make sure that they have writing rights on the selected file, and also that the content of that file is not needed any more, as it will be deleted by StdpC as soon as the clamping cycle begins. The rectangular button on the right of the text line allows for file browsing.
- Saving frequency: sets the frequency of the data saving.
- Saving format: this combo allows for choosing between ASCII and binary output format.

11 Input channel configuration

The *Input Channels* dialog contains the basic configurable properties for the input channels, like whether they are going to be active or not, their acquisition range and conversion factor, along with some more StdpC specific properties, like whether spike detection is going to be on or off on them (see Section 7.10), and if it is on, what spike detection threshold will be applied, and finally whether the channel is going to be saved or not during dynamic clamping (Section 10). Always make sure that the right conversion factor is applied for each active channel, and a sufficiently wide acquisition range is set in order to prevent out of range data being cut off of the recorded data.

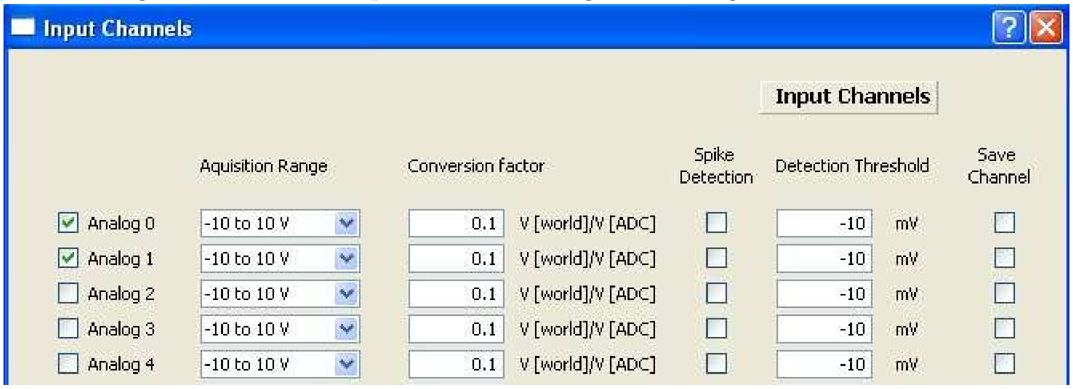

12 Output channel configuration

The *Output Channels* dialog contains the basic configurable properties for the output channels, like whether they are going to be active or not, their acquisition range and conversion factor, what is the bias current level applied on that channel if any, and finally whether the channel is going to be saved or not during dynamic clamping (Section 10). Always make sure that the right conversion

factor is applied for each active channel, and a sufficiently wide acquisition range is set in order to prevent out of range values being cut off of the injected current.

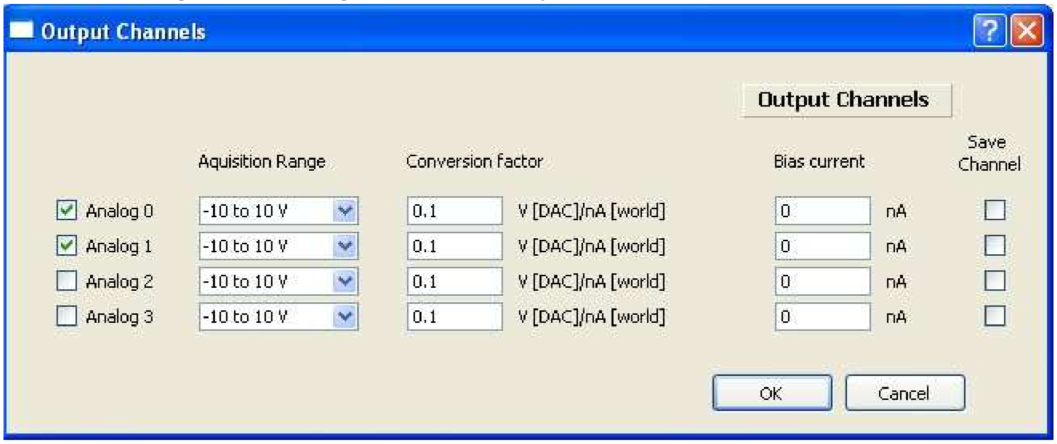

13 User feedback

Please do give feedback if you found the software useful, buggy, or have general comments or questions. Please send correspondence through the sourceforge email or directly to T.Nowotny@sussex.ac.uk. If you are interested in contributing to the future development please do not hesitate to contact me as well.

14 Acknowledgements

The StdpC software would not exist without the early dynamic clamp versions developed by Reynaldo D. Pinto and I (TN) am grateful to him for supplying the source code for further development back then. I am also grateful to Attila Szücs for his excellent suggestions for improving and extending the software and his tireless testing of new and buggy versions.

References

Abarbanel, H. D. I., Huerta, R., and Rabinovich, M. I. (2002). Dynamical model of longt-term synaptic plasticity. *P Natl Acad Sci USA*, 99:10132–10136.

Brette, R., Piwkowska, Z., Monier, C., Rudolph-Lilith, M., Fournier, J., Levy, M., Frégnac, Y., Bal, T., and Destexhe, A. (2008). High-resolution intracellular recordings using a real-time computational model of the electrode. *Neuron*, 59(3):379–391.

Destexhe, A., Mainen, Z. F., and Sejnowski, T. J. (1994). An efficient method for computing synaptic conductances based on a kinetic model of receptor binding. *Neural Comput*, 6:14–18.

Hodgkin, A. L., Huxley, A. F., and Katz, B. (1949). Ionic current underlying activity in the giant axon of the squid. *Arch Sci Physiol*, 3:129–150.

Kemenes, I., Marra, V., Crossley, M., Samu, D., Staras, K., Kemenes, G., and Nowotny, T. (2011). Dynamic clamp with stdpc software. *Nature Protocols*, 6(3):405–417.

Nowotny, T., Szucs, A., Pinto, R. D., and Selverston, A. I. (2006). Stdpc: a modern dynamic clamp. *J Neurosci Methods*, 158(2):287–299.

Pinto, R. D., Elson, R. C., Szücs, A., Rabinovich, M. I., Selverston, A. I., and Abarbanel, H. D. I. (2001). Extended dynamic clamp: Controlling up to four neurons using a single desktop computer and interface. *J Neurosci Meth*, 108:39–48.

Rall, W. (1967). Distinguishing theoretical synaptic potentials computed for different soma-dendritic distributions of synaptic inputs. *J Neurophysiol*, 30:1138–1168.

Robinson, H. P. and Kawai, N. (1993). Injection of digitally synthesized synaptic conductance transients to measure the integrative properties of neurons. *J Neurosci Meth*, 49:157–165.

Sharp, A. A., O'Neil, M. B., Abbott, L. F., and Marder, E. (1993). Dynamic clamp: computer-generated conductances in real neurons. *J Neurophysiol*, 69:992–995.

Traub, R. D. and Miles, R. (1991). *Neural Networks of the Hippocampus*. Cambridge University Press, New York.
